# 3D‐Zipped Interface: In Situ Covalent‐Locking for High Performance of Anion Exchange Membrane Fuel Cells

**DOI:** 10.1002/advs.202102637

**Published:** 2021-10-11

**Authors:** Xian Liang, Xiaolin Ge, Yubin He, Mai Xu, Muhammad A. Shehzad, Fangmeng Sheng, Rachida Bance‐Soualhi, Jianjun Zhang, Weisheng Yu, Zijuan Ge, Chengpeng Wei, Wanjie Song, Jinlan Peng, John R. Varcoe, Liang Wu, Tongwen Xu

**Affiliations:** ^1^ CAS Key Laboratory of Soft Matter Chemistry Collaborative Innovation Center of Chemistry for Energy Materials Department of Applied Chemistry School of Chemistry and Materials Science University of Science and Technology of China 96 Jinzhai Road Hefei Anhui 230026 P. R. China; ^2^ School of Chemistry and Material Engineering Huainan Normal University Huainan Anhui 232001 P. R. China; ^3^ Department of Chemistry University of Surrey Guildford Surrey GU2 7XH UK; ^4^ The Center for Micro‐ and Nanoscale Research and Fabrication University of Science and Technology of China 96 Jinzhai Road Hefei Anhui 230026 P. R. China

**Keywords:** catalyst layers, fuel cells, membrane electrode assembly, interfaces, ionomers

## Abstract

Polymer electrolyte membrane fuel cells can generate high power using a potentially green fuel (H_2_) and zero emissions of greenhouse gas (CO_2_). However, significant mass transport resistances in the interface region of the membrane electrode assemblies (MEAs), between the membrane and the catalyst layers remains a barrier to achieving MEAs with high power densities and long‐term stabilities. Here, a 3D‐interfacial zipping concept is presented to overcome this challenge. Vinylbenzyl‐terminated bi‐cationic quaternary‐ammonium‐based polyelectrolyte is employed as both the anionomer in the anion‐exchange membrane (AEM) and catalyst layers. A quaternary‐ammonium‐containing covalently locked interface is formed by thermally induced inter‐crosslinking of the terminal vinyl groups. Ex situ evaluation of interfacial bonding strength and in situ durability tests demonstrate that this 3D‐zipped interface strategy prevents interfacial delamination without any sacrifice of fuel cell performance. A H_2_/O_2_ AEMFC test demonstration shows promisingly high power densities (1.5 W cm^−2^ at 70 °C with 100% RH and 0.2 MPa backpressure gas feeds), which can retain performances for at least 120 h at a usefully high current density of 0.6 A cm^−2^.

## Graphical abstract

A 3D‐interfacial zipping design is reported to in situ fabricate interfacial covalently locked membrane electrode assembly by thermally induced inter‐crosslinking of the terminal vinyl groups of the ionomer in the catalyst layers and the anion‐exchange membrane. Ex situ evaluation of interfacial bonding strength and in situ durability tests demonstrate that the 3D‐zipped interface strategy prevents interfacial delamination without any sacrifice of fuel cell performance.

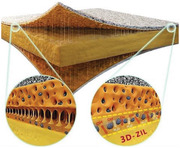

## 1 Introduction

Polymer electrolyte membrane fuel cells, including proton exchange membrane fuel cells (PEMFCs) and anion exchange membrane fuel cells (AEMFCs), have made significant strides in the highly efficient and sustainable production of electrical power.^[^ ^1^, ^2^, ^3^, ^4^ ^]^ For PEMFCs, the use of perfluorosulfonic acid (PFSA) polymers for the preparation of membrane electrode assemblies (MEAs) has resulted in PEMFC‐equipped commercial vehicles.^[^ ^5^, ^6^, ^7^, ^8^, ^9^, ^10^ ^]^ In the past few years, AEMFCs have made great breakthroughs in achieving high performances with peak power densities (PPDs) now common above 1 W cm^–2^ (Fuel cell data of recently reported anionomers (also known as anion exchange ionomers, AEIs) or anion exchange membrane (AEMs) for H_2_/O_2_ AEMFCs represented in Table S1, Supporting Information).^[^ ^11^, ^12^, ^13^, ^14^, ^15^, ^16^, ^17^, ^18^, ^19^, ^20^, ^21^, ^22^, ^23^, ^24^, ^25^, ^26^, ^27^, ^28^, ^29^, ^30^, ^31^, ^32^, ^33^, ^34^ ^]^ These studies are gradually narrowing the performance gap with PEMFCs due to the development of AEMs accompanied by high OH^−^ conductivity as well as optimization of catalytic electrode designs and water balance. However, the lack of long‐term AEMFC durabilities, particularly at high temperatures, low humidities, and/or with realistic current density operation, is a critical barrier to adoption into commercial markets (cf. PEMFCs).^[^ ^35^ ^]^ This has spurred the recent, intense effort into the development of AEIs and AEMs both with the competitive OH^−^ conductivities and alkaline stabilities that has led to a notable improvement in operational H_2_/O_2_ AEMFC durabilities.^[^ ^36^, ^37^, ^38^, ^39^, ^40^, ^41^, ^42^, ^43^, ^44^ ^]^ However, AEMFC durabilities that are high enough for commercial deployment is still far away compared to PEMFCs.^[^ ^14^, ^15^ ^]^

In this context, this critical limitation is not exclusively due to the materials making up the MEAs, but also due to a poor understanding and control over the nature of the interface between the AEM and AEI‐containing catalyst layers (CLs) in the MEAs. The effect of MEA composition and structure on the transport properties of reactant gases (H_2_ or O_2_), OH^−^ ions, and H_2_O molecules ultimately dictates AEMFC performance.^[^ ^45^, ^46^, ^47^ ^]^ Among these, the interfacial contact between membrane and CLs will play an essential part.^[^ ^48^, ^49^ ^]^ In the AEMFC, four H_2_O molecules are produced at the anode for every four electrons transferred, with consumption of two H_2_O molecules at the cathode. This can create a large water gradient between electrodes with undesirable flooding in the anode and drying out (chemical stability problems) in the cathode, without adequate back‐diffusion of H_2_O from anode to cathode (with the use of thin, high diffusivity AEMs).^[^ ^50^, ^51^ ^]^ Our previous work showed that a significant water gradient causes excessive interface delamination between AEM and the anode CL,^[^ ^52^ ^]^ leading to increased OH^−^ transport resistances and a rapid decline in AEMFC durability. In a PEMFC, water flooding can be mitigated because only two water molecules are electrochemical produced (at the cathode) for every four electrons transferred.^[^ ^50^ ^]^ Therefore, the difference in water environment between AEMFCs and PEMFCs will accelerate the delamination of AEM‐CLs interface. Hence, Kim, et al. proposed that membrane–electrode interfacial compatibility is a critical issue in PEMFCs and AEMFCs. Fuel cell performance degradation scaled well with initial membrane–electrode interfacial resistance, suggesting that the membrane–electrode interface had a tremendous impact on fuel cell durability.^[^ ^36^, ^53^, ^54^ ^]^ Furthermore, the use of PFSA copolymers (as PEM and ionomer in the CLs), which are basically the same class of polymer and have relatively low glass transition temperature (*T*_g_), facilitates a flexible thermal lamination process to suppress problems with the interfacial delamination.^[^ ^6^ ^]^ However, the AEMFCs commonly use hydrocarbon AEMs and AEIs that commonly have a high *T*_g_, which leads to difficulty in achieving adequate interfacial bonding strength (thermal lamination of the AEM to CLs requires high temperatures that risks AEM/AEI chemical degradation).

**Figure 1:**
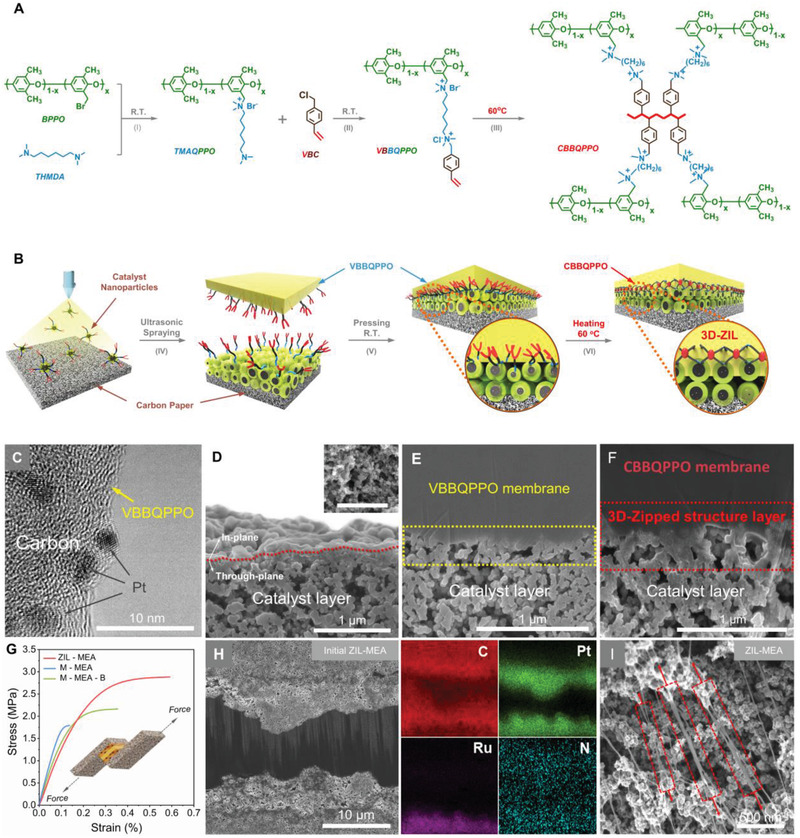
Schematic of the 3D covalently locked interface strategy and micromorphology at different stages for fabrication of durable ZIL‐MEA. A) Chemical scheme of the synthesis and inter‐crosslinking of VBBQPPO. B) Preparation of the 3D‐zipped interface layer through the thermal inter‐crosslinking between the terminal vinyl groups of the VBBQPPO polymer located in the CLs and AEM. C) TEM image of the VBBQPPO@Pt/C catalyst nanoparticles. D) SEM image of the CL of the GDE (the inset is the SEM surface image of the CL, and the scale of the inset is 1 µm). E) SEM cross‐sectional image of the MEA, which contains VBBQPPO membrane and VBBQPPO@metal/C catalyst layer, after lamination at room temperature. F) SEM cross‐sectional image of MEA after thermally triggered covalent crosslinking between the AEM and CL. G) Tensile stress–strain curves for shear testing of the dry ZIL‐MEA, the dry M‐MEA (pre‐crosslinked benchmark), and the dry M‐MEA‐B (uncrosslinked benchmark); H) cross‐section SEM coupled with EDX images of initial ZIL‐MEA. I) CBBQPPO Pt/C CL surface of the ZIL‐MEA after the shear test, with highly deformed AEIs fibers. Commercial Pt/C and PtRu/C electrocatalysts were used in the cathode and anode, respectively. To ensure the consistency the of the data, SEM images of the CL were of the VBBQPPO@Pt/C catalyst layer (cathode), unless otherwise specified.

We therefore propose that a more intelligent design of a stable AEM‐electrode interface is required for AEMFCs containing hydrocarbon electrolyte components. Rather than using the conventional strategy (increasing physical friction of the interface),^[^ ^55^, ^56^, ^57^, ^58^, ^59^ ^]^ in this study, we propose a thermally triggered interlocking strategy to obtain a tightly bound interface via chemical bonding of the hydrocarbon AEM and hydrocarbon AEIs in the CLs of the electrodes.^[^ ^60^, ^61^ ^]^ A quaternary ammonium‐(QA)‐functionalized polyelectrolyte, containing pendant chains with terminal vinyl groups, was synthesized for the AEM and AEI used in the CLs (**Figure** **1**A). A N_2_/N_2_ in situ operation of the resulting MEA lead to inter‐crosslinking of the terminal vinyl groups, forming a covalently locked interface (this MEA is designated ZIL‐MEA). This QA‐containing interface provides both ionically conductive interconnections and prevents interfacial delamination during wet/dry cycling. Other two MEAs containing individually pre‐crosslinked AEM and gas diffusion electrodes (GDEs) (or uncrosslinkable AEM and GDEs) were fabricated as the non‐interfacial‐locked benchmarks (designated as M‐MEA or M‐MEA‐B). The importance of the covalently locked interface in stabilizing the MEA, while still allowing high power outputs is discussed below.

## 2 Results and Discussion

### 2.1 Synthesis for the Crosslinkable Polymer Precursor

The vinylbenzyl‐terminated bi‐cationic quaternary ammonium‐poly(2,6‐dimethylphenylene oxide)‐PPO (VBBQPPO) AEI was synthesized in two steps (Figure 1A and Scheme S1, Supporting Information). The polymer precursor tertiary‐amine‐terminated quaternary ammonium‐PPO (TMAQPPO) was synthesized via the Menshutkin reaction between the bromomethyl groups of bromomethylated PPO (BPPO) (^1^H NMR spectra shown in Figure S1, Supporting Information) and tertiary amine groups of *N*,*N*,*N′′*,*N′′*‐tetramethyl‐1,6‐hexanediamine (TMHDA) (procedure represented in Figure 1A(I), and ^1^H NMR spectra shown in Figure S2, Supporting Information). The terminal tertiary amine groups further reacted with the —CH_2_Cl of the 1‐(chloromethyl)‐4‐ethenyl‐benzene (VBC) to obtain VBBQPPO (the procedure represented in Figure 1A(II)). The presence of vinyl proton signals in ^1^H NMR spectra (*δ*_H_ = 5.35, 5.85, and 6.75 ppm) demonstrate that the Menshutkin reaction proceeded successfully with the vinyl groups remaining unreacted (Figure S3, Supporting Information).^[^ ^62^, ^63^ ^]^ As a comparison, the uncrosslinkable bi‐cationic quaternary ammonium‐based PPO (BQPPO) was synthesized via the Menshutkin reaction between BPPO and 6‐(dimethylamino)‐*N*‐ethyl‐*N*,*N*‐dimethylhexan‐1‐aluminum bromide (DMAQA, synthesized by one‐sided quaternization of TMHDA) (Scheme S2, Supporting Information).^[^ ^64^ ^] 1^H NMR spectra of DMAQA and BQPPO are shown in Figures S4 and S5, Supporting Information, respectively. The ion‐exchange capacity (IEC) of crosslinked vinylbenzyl‐terminated bi‐cationic quaternary ammonium PPO (CBBQPPO) and BQPPO was both controlled at 2.00 ± 0.02 mmol g^–1^.

In situ FT‐IR analysis shows that the terminal vinyl groups are capable of thermally initiated inter‐crosslinking (conversion of VBBQPPO into CBBQPPO shown in Figure 1A(III)). This reaction occurs gradually on increasing the temperature from 30 °C to 70 °C (Figure S6, Supporting Information). The intensity of the C═C stretching vibration at 1610 cm^–1^ decreases with increasing temperature (Figure S6A, Supporting Information), while the intensity of the overlapping C—H vibrations at 2900–3020 cm^–1^ increases due to the methylene and methine groups generated during the thermal crosslinking reaction (Figure S6B, Supporting Information). The thermally initiated crosslinking process was verified by studying the difference in the solubility of VBBQPPO and CBBQPPO in *N*‐methylpyrrolidone (NMP). CBBQPPO exhibits poor solubility, accompanied by the formation of colloid, compared to the VBBQPPO AEI (Figure S6C,D). Therefore, the thermal lamination of corresponding MEA, using VBBQPPO as both AEM and AEI in the CLs, is hypothesized to form an interlocked interface for enhanced fuel cell durability (Figure 1B(VI)). Additionally, in the case of similar OH^‐^ conductivity at different temperature (30–80 °C, Figure S7A), water uptake (Figure S7B, Supporting Information) and swelling ratio (Figure S7C, Supporting Information) of CBBQPPO are lower than that of BQPPO, indicating that CBBQPPO has better dimensional stability to maintain the structural stability of the MEA. Evidence in support of this hypothesis is discussed in the following sections.

### 2.2 Fabrication of MEA with the 3D‐Zipped Interface Layer

Each MEA was fabricated by sandwiching a VBBQPPO‐based AEM between two VBBQPPO‐treated CLs (containing commercial Pt/C and PtRu/C electrocatalysts at the cathode and anode, respectively, as these are the most commonly reported electrocatalysts and this is concerned with the feasibility of the thermally initiated covalent‐locking strategy). The VBBQPPO‐catalyst dispersions were fabricated by ultrasonically dispersing the catalysts in a solution (mixed water and isopropyl alcohol) of VBBQPPO. Transmission electron microscopy (TEM) images of the VBBQPPO‐Pt/C dispersion shows that a thin AEI shell (amorphous feature without obvious lattice fringes) uniformly envelope the Pt/C nanoparticles (NP) (Figure 1C and Figure S8A, Supporting Information). The highly solvated VBBQPPO@Pt/C dispersion was sprayed onto gas diffusion layer (GDL) by inkjet‐printing at room temperature to fabricate a GDE precursor (the procedure presented in Figure 1B(IV)). The atomic force microscope (AFM) phase image (Figure S8B, Supporting Information) and X‐ray photoelectron spectroscopy (XPS) spectrum (Figure S8C, Supporting Information) further demonstrate the uniform loading of the AEI on the catalyst surface. Notably, the catalyst nanoparticles (NPs, blue‐purple) were uniformly dispersed and connected within the AEI phase (yellow) to form a porous morphology (Figure S8B, Supporting Information), which provides a significant three‐phase reaction interface for electrode reactions. In addition, the peak at 402.25 eV in XPS spectrum represents N 1s, which is assigned to the C—N^+^ species in the QA groups of VBBQPPO, providing initial evidence of VBBQPPO polymer‐encapsulated catalysts (Pt/C NPs). Meanwhile, the VBBQPPO AEM (15 µm thickness, 2.00 ± 0.02 mmol g^–1^ of IEC) was prepared by the solution casting method. The VBBQPPO‐based MEA was fabricated by assembling the AEM between the VBBQPPO@Pt/C cathode and VBBQPPO@PtRu/C anode GDEs at room temperature (the procedure presented in Figure 1B(V)). The low fabrication temperature did not initiate the self‐polymerization of the vinyl functionalities of VBBQPPO because a loose boundary interface was clearly observed in the scanning electron microscopy (SEM) cross‐sectional image of the MEA (Figure 1E and Figure S9A, Supporting Information).

An AEMFC single cell was fabricated by assembling the MEA between two graphite bipolar plates at anode and cathode. To in situ convert VBBQPPO‐based MEA into crosslink‐immobilized form (CBBQPPO@Pt/C, CBBQPPO@PtRu/C, and CBBQPPO AEM), the cell operating temperature was increased from 30 to 60 °C over 30 min and then maintained at 60 °C for 2 h, with N_2_ gas provided to both the cathode and anode (flow rate of 0.5 L min^–1^, 100% RH). The terminal vinyl groups in the MEA undergo a thermally initiated inter‐crosslinking reaction by this pretreatment, with the reaction between the VBBQPPO‐based AEM and CLs (containing VBBQPPO AEI) yielding a covalently locked zipped interface. This converts the weak physical contact interface into a dense 3D‐zipped interface layer (3D‐ZIL) with a thickness of 100–200 nm (Figure 1E,F and Figure S9B, Supporting Information). The SEM images also indicate that the inter‐crosslinking does not sacrifice the porous geometry of the CLs, thus facilitating efficient gas and ions transport during fuel cell operation.

Cross‐sectional SEM and energy‐dispersive X‐ray (EDX) spectrometry analysis provides further evidence of the thermally triggered formation of 3D‐ZIL. The VBBQPPO‐based MEA with 3D‐ZIL (ZIL‐MEA) exhibits a tight chemical contact between the AEM and CLs (Figure 1H). In contrast to the EDX mapping of the distribution of Pt and Ru atoms within distinct boundary regions, N atoms were uniformly distributed throughout the cross‐section of the ZIL‐MEA. This supports the formation of integrated MEA with the 3D‐ZIL after the occurrence of the interfacial inter‐crosslinking reaction, in line with our aim of preventing the delamination of the MEA during cell discharge. Without 3D‐Zipped interface, M‐MEA (precrosslinked benchmark) and M‐MEA‐B (uncrosslinked benchmark) were fabricated for comparison purposes: especially in M‐MEA, the VBBQPPO‐containing AEM and GDEs were precrosslinked at 60 °C (CBBQPPO‐based) prior to MEA assembly using the same procedure as for ZIL‐MEA.

The interfacial bonding strength for a dry ZIL‐MEA and a dry M‐MEA/M‐MEA‐B were compared (Figure 1G and Table S2, Supporting Information). The average interfacial bonding strength was 0.79 N mm^−1‐^ for M‐MEA or 1.02 N mm^−1‐^ for M‐MEA‐B, which was significantly higher for ZIL‐MEA (1.30 N mm^−1^). This demonstrates the robustness of the covalently locked ZIL structure. When the interface layer tears on application of tension at both sides of the MEA, the AEI in the CLs is expected to deform (with stretching due to its rubber‐like character). Changes in the internal morphology of the CLs were probed using SEM analysis both before and after the shear test (Figure 1I and Figure S8D–F, Supporting Information). The initial surface porous geometry of VBBQPPO@Pt/C CL is shown in Figure S8D, Supporting Information. The SEM image of the fractured CL surface for the ZIL‐MEA shows AEI phase is severely deformed into filaments due to energy dissipation during delamination (Figure 1I and Figure S11, Supporting Information), with retention of bonding to the AEM. This fracture behavior contrasts with that of M‐MEA (Figure S8F, Supporting Information, post‐shear test), where the delaminated CBBQPPO@Pt/C CL retains a porous morphology that appears similar to the initial surface (Figure S8D, Supporting Information) and with no evidence of filament formation. Therefore, the 3D‐ZIL concept facilitates the dissipation of stress. The applied force is distributed throughout the 3D‐ZIL, giving rise to a considerably enlarged energy dissipation zone (cf. M‐MEA or M‐MEA‐B).

It is well known a major driving force for interfacial delamination within MEAs during fuel cell operation is the tension generated at the interface between the membrane and CLs due to dissimilar extents of component volume expansions upon in situ variations in hydration.^[^ ^30^, ^31^ ^]^ Therefore, the uniformly enveloped AEI shells within a flexible 3D‐ZIL is expected to suppress the in situ interfacial delamination of the MEA during fuel cell operation.

### 2.3 Fuel Cell Performance

**Figure 2:**
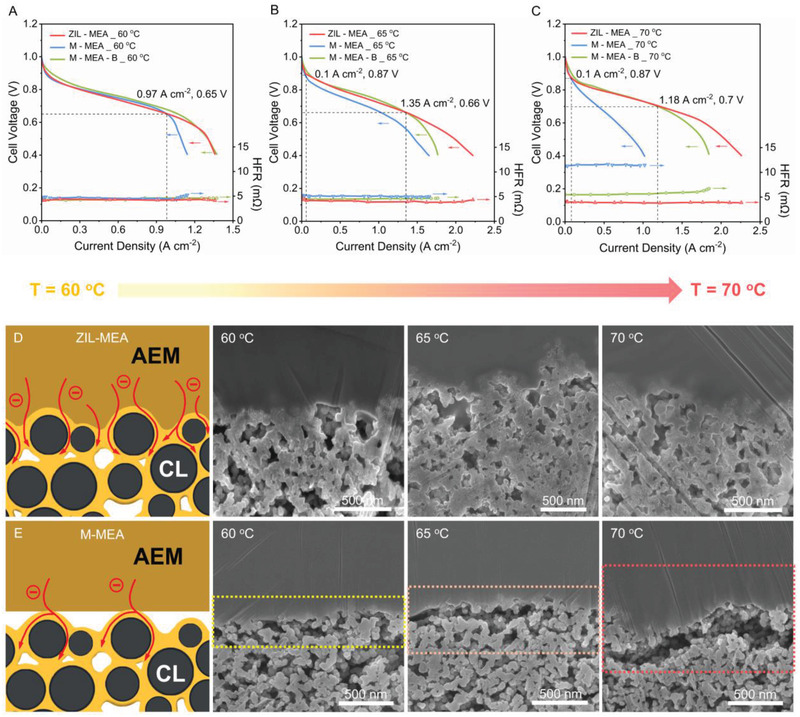
Single‐cell H_2_/O_2_ AEMFC performance data at 60, 65, and 70 °C for ZIL‐MEA, M‐MEA, and M‐MEA‐B with the corresponding cross‐sectional SEM images of the post‐test interfaces. A–C) Polarization curves and HFR data (geometric electrode area 12.25 cm^2^) at the indicated test temperatures. D) Schematic for the OH^−^ ion conduction pathway within the ZIL‐MEA and the cross‐sectional SEM images of cathode side of the ZIL‐MEA after AEMFC testing at the indicated temperatures. E) Schematic for the OH^−^ ion conduction pathway within the M‐MEA and the cross‐sectional SEM images of cathode side of the M‐MEA after AEMFC testing at the indicated temperatures. The AEI content for all CLs was 20 wt%. Electrocatalysts were used with a metal loading of 0.5 mg_metal_ cm^−2^ (PtRu/C in anode and Pt/C in cathode). The flow rate of all gases was controlled at 0.5 L min^−1^.

Even though ZIL‐MEA exhibits high interfacial bonding strength in ex situ shear tests, to estimate its application potential, an initial in situ H_2_/O_2_ AEMFC performance evaluation is necessary. After assembly between two graphite bipolar plates, the ZIL‐MEA was activated under potentiostatic control (constant cell discharge of 0.50 V at 60 °C) until a stable power density was achieved. The benchmark single cell containing the M‐MEA or M‐MEA‐B was fabricated and activated using the same procedure. Fuel cell performances of the three MEAs were characterized at 60, 65, and 70 °C. Figure S12 (Supporting Information) presents the PPD and corresponding high‐frequency resistance (HFR) data as a function of temperature for three types of MEA. The ZIL‐MEA exhibits an increasing PPD with temperature, whereas the performance of M‐MEA declines sharply at a high temperature (no increase for M‐MEA‐B after 65 °C) due to increased mass transport resistance. As shown in Figure S13A, Supporting Information, three MEAs have comparable single cell performance at 60 °C (PPD of ZIL‐MEA is 695 mW cm^–2^, PPD of M‐MEA‐B is 700 mW cm^–2^, and PPD of M‐MEA is 649 mW cm^–2^). The numerical discrepancy in PPD (Δ*P*_60_) between ZIL‐MEA and M‐MEA is 46 mW cm^–2^, and which between ZIL‐MEA and M‐MEA‐B (Δ*P*_65_) is only 5 mW cm^–2^. The corresponding polarization curves in **Figure** **2**A show that the performance difference is mainly derived from an increase in HFR at current densities above 0.97 A cm^–2^ with the M‐MEA. An increase in temperature should lead to better oxygen reduction reaction (ORR) and hydrogen oxidation reaction (HOR) kinetics. Hence, raising the operating temperature to 65 °C leads to an increased PPD with the ZIL‐MEA (increases to 1008 mW cm^–2^). However, the PPD of M‐MEA and M‐MEA‐B did not increase to such a large extent (761 mW cm^–2^ for M‐MEA and 926 mW cm^–2^ for M‐MEA‐B), leading to Δ*P*_65_ being an increased 247 mW cm^–2^ and Δ*P*′_65_ being an increased 82 mW cm^–2^ (Figure S13B, Supporting Information). Notably, Δ*P*_70_ increased sharply to 642 mW cm^–2^ (Δ*P*′_70_ increased to 137 mW cm^–2^) at 70 °C due to the rapidly increased HFR of M‐MEA and M‐MEA‐B (Figure 2C and Figure S13C, Supporting Information). ZIL‐MEA maintains a low HFR over the entire AEMFC test temperature range (60–70 °C) due to the stable 3D‐ZIL (see cartoon in Figure 2D), which sustains facile OH^−^ transport between the AEM and CLs. On the contrary, the degradation in AEMFC performance at higher temperatures with the M‐MEA or M‐MEA‐B supports the ex situ data where MEA delamination is more likely (especially at high current density due to increased water production at the anode) (Figure 2E). Changes in the internal resistance (IR) of these three MEAs, estimated by the slope of the Ohmic region (slope = Δ*V*/Δ*I*, mΩ) in the polarization curves (Figure S13D–F, Supporting Information), provides further evidence that the influence of 3D‐ZIL on the fuel cell performance is mainly due to mass transport resistance. The IR of ZIL‐MEA gradually decreases during AEMFC test (19 mΩ, 60 °C; 16 mΩ, 65 °C; 11 mΩ, 70 °C) with slowly IR decrease of M‐MEA‐B (19 mΩ, 60 °C; 17 mΩ, 65 °C; 14 mΩ, 70 °C), while the IR of M‐MEA increases sharply (19 mΩ, 60 °C; 19 mΩ, 65 °C; 33 mΩ, 70 °C). Especially, ZIL‐MEA shows a competitive PPD with 0.2 MPa backpressure gas feeds (1.5 W cm^−2^ at 70 °C with 100% RH, shown as Figure 5A), which is the highest reported for PPO‐based polymer materials used in AEMFCs (Table S1, Supporting Information).

To further prove this result, SEM investigations were used to detect morphological changes in the AEM‐CL interfaces of both MEAs during the AEMFC operation (Figure 2D,E). ZIL‐MEA maintains its tight contact interface during AEMFC testing (the duration of tests being ≈10 h) at the higher operating temperatures. In contrast, higher temperature degrades the interface integrity in M‐MEA due to strong rigidity of the cross‐linked AEM,^[^ ^65^ ^]^ resulting in clear delamination. This result is consistent with the change in Δ*P* and HFR data discussed above. Considering both ZIL‐MEA and M‐MEA possessed the same catalysts, AEIs and AEM, the significant difference in AEMFC performances must come mainly because of the difference in the AEM‐CL interfaces. The stable 3D‐ZIL, with reduced mass transport and ohmic losses, facilitates high AEMFC performance, especially at higher temperatures and discharge current densities. The strategy tested (in situ 3D‐ZIL formation) does protect the structural integrity of the MEA to help maintain high performances.

### 2.4 Durability Evaluation at a Constant and Shifted Relative Humidity

**Figure 3:**
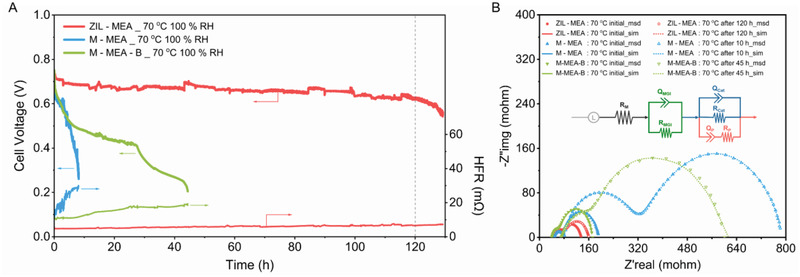
Initial constant RH durability testing. A) H_2_/O_2_ AEMFCs durability evaluation results with ZIL‐MEA, M‐MEA, and M‐MEA‐B at 70 °C and 100% RH (cell maintained at a constant 0.6 A cm^−2^ cell discharge). B) In situ EIS conducted on the ZIL‐MEA, M‐MEA, and M‐MEA‐B before and after the constant current discharge test. The full equivalent circuit diagram can be expressed as *LR*_M_(*Q*_MGI_ *R*_MGI_)(*Q*_cat_ *R*_cat_(*Q*_P_ *R*_P_)), where the contribution of the electrically conductive fuel cell components (including electrodes) was modeled by the inductance element (*L*) and *R*_M_ represents the ohmic resistance of the AEM. The first circuit, (*Q*_MGI_ *R*_MGI_) attributes the charge‐storage (*Q* = constant phase element) and charge‐transfer resistance (*R*) between the MGI. The second circuit, (*Q*_cat_ *R*_cat_(*Q*_P_ *R*_P_)) represents the charge‐storage and charge‐transfer resistance values at the catalyst–polymer interface within the GDE. Details of the measured (msd) and best‐fit simulated (sim, equivalent electrical circuit model) EIS–Nyquist data are given in Table S3, Supporting Information (insert, error <0.5%).

To further investigate the promise of the 3D‐ZIL concept, initial durability testing of both MEAs was conducted by discharging each benchmark in an H_2_/O_2_ AEMFC at a current density of 0.6 A cm^–2^ at 70 °C with 100% RH (no backpressure) gas supplies (**Figure** **3**A). In contrast to the ZIL‐MEA, a sharp drop in cell voltage from 0.73 to <0.25 V (concurrent with the rapid rise of HFR) was observed for the benchmarks M‐MEA‐B and M‐MEA, which is consistent with the results of the interfacial shear test. Although chemical degradation has an important impact on MEA failure, considering that CBBQPPO and BQPPO have simultaneous degradation in alkaline environment,^[^ ^66^, ^67^ ^]^ comparable OH^‐^ conductivity (Figure S7A, Supporting Information) and hydroxide stability (Figure S7D, Supporting Information), the worse durability of M‐MEA (than that of M‐MEA‐B) was mainly caused by the faster interfacial delamination due to high rigidity of CBBQPPO. Our recent study has proven that the AEMFC performance attenuation related to the increase of AEM resistance (*R*_M_) and contact resistances (*R*_c_ = *R*_MGI_ + *R*_cat_ + *R*_P_) in the MEA both before and after the durability evaluation test (RMGI refers to the charge‐transfer resistance (*R*) between the membrane‐GDE interface (MGI), while “*R*_cat_ + *R*_p_” refer to the charge‐transfer resistance values at the catalyst–polymer interface within the GDE; quantified data are given in Table S3, Supporting Information, the equivalent circuit model is shown in Figure 3B).^[^ ^30^ ^]^ The simulated electrochemical impedance spectroscopy (EIS) data for M‐MEA demonstrates a 65% increase in *R*_M_ (71% for M‐MEA‐B), a 34% increase in *R*_cat_ (59% for M‐MEA‐B), but a substantial 930% increase in *R*_MGI_ (579% for M‐MEA‐B). In contrast, a negligible 18.8% increase in *R*_MGI_ (6.6 mΩ increase) is observed for the ZIL‐MEA, again supporting negligible in situ interfacial delamination with the ZIL‐MEA. IR and HFR data during fuel cell test and the impedances proposed in EIS analysis both prove that the 3D‐ZIL is essential to the stable and efficient transport of ions between the polymer electrolyte components in the cell.

**Figure 4:**
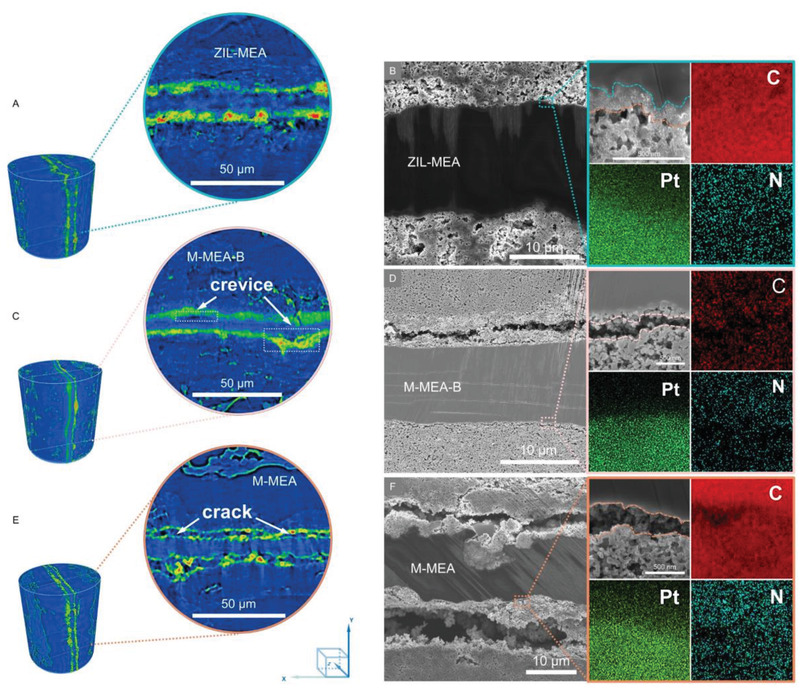
The resulting changes in the MEAs after initial constant RH durability testing. A,C,E) Micro‐CT images of ZIL‐MEA (A), M‐MEA‐B (C), and M‐MEA (E) and B,D,F) cross‐section SEM images coupled with EDX for ZIL‐MEA (B), M‐MEA‐B (D), and M‐MEA (F), all recorded after the constant current discharge durability test.

Furthermore, the morphological changes at the AEM‐CL interface in the MEAs can also be matched with the simulated EIS data (Table S3, Supporting Information) and the interfacial bonding strength (Figure 1G). Compared with the initial cross‐sectional morphology of M‐MEA‐B and M‐MEA (Figures S9 and S10, Supporting Information), due to the formation of crevice (Figure S16, Supporting Information, high‐magnification images of **Figure** **4**D,F), the physical contact interface in M‐MEA‐B or M‐MEA was observed to crack during durability test, which correlates with the decline in AEMFC performance characteristics discussed above. In contrast, the ZIL‐MEA showed that 3D‐ZIL again maintained a tight connection between the AEM and CL (Figure 4B). More detailed SEM imaging of the post‐durability test morphology for the ZIL‐MEA is presented in Figure S14, Supporting Information. Moreover, changes in the internal structure of the MEAs were investigated using computed micro‐X‐ray tomography.^[^ ^68^ ^]^ Benefiting from the 3D‐ZIL, even after 120 h durability test, the AEM and CLs in ZIL‐ MEA remain tightly bonded with no visible cracks in any position through a 3D reconstruction of the MEA sample volume dataset (Figure 4A; VideosS1 and S2, Supporting Information). While the ZIL‐MEA maintains integrity, the M‐MEA clearly experienced delamination events during the durability test (Figure 4E). Meanwhile, due to the better molecular chain flexibility of BQPPO (than CBBQPPO), the M‐MEA‐B partially experienced delamination (Figure 4C).

**Figure 5:**
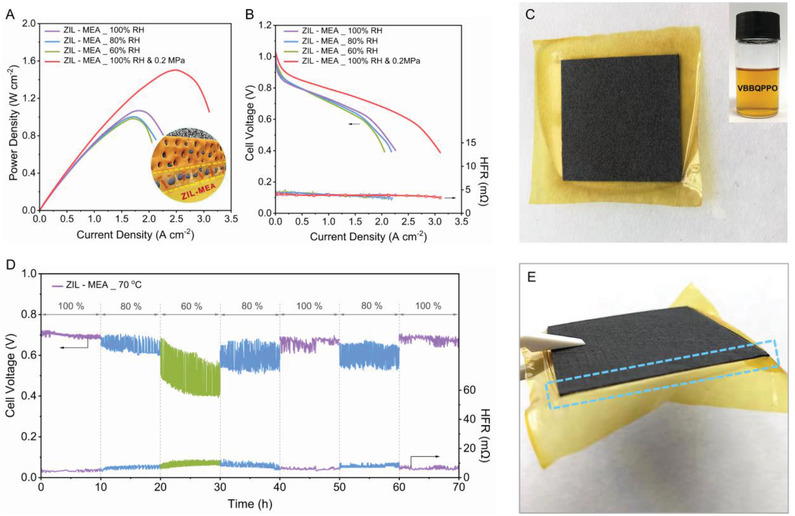
An initial RH cycling durability evaluation. A,B) H_2_/O_2_ AEMFC performance data at 70 °C (electrode area 12.25 cm^2^) for the ZIL‐MEA with different RHs (60–100%) gas supplies. C,E) The macroscopic optical appearance of the front (C) (the inset is VBBQPPO ionomer solutions in ethanol) and profile (E) of ZIL‐MEA after 2 days of AEMFC RH cycling. D) RH cycling of the ZIL‐MEA during H_2_/O_2_ AEMFC discharge at 0.6 A cm^–1^. The detailed RHs, backpressures of the anode and cathode gas are same in all AEMFC performance tests.

AEMFC performance and durability can be limited by the water differential across the MEA, with the potential for undesirable flooding in the anode and dry out of the cathode. **Figure** **5**A shows H_2_/O_2_ AEMFC performance of the ZIL‐MEA at 70 °C with different RH gas supplies. Only a minor performance loss was observed with a drop of RH to 60%, while the HFR showed very little variation (Figure 5B); this illustrating that no mechanical degradation occurs on changes of RH due to the tight contacts (by design) at the interface between the AEM and CLs.

To further probe this RH resilience, the durability of the ZIL‐MEA‐based H_2_/O_2_ AEMFC was studied during RH cycling (Figure 5D). The ZIL‐MEA maintained cell voltage and HFR in the initial 10 h with 100% RH gases supplies to both the anode and cathode. Voltage fluctuations (≈0.1 V amplitude) were observed during the subsequent 10 h when the RH was lowered to 80%. However, as the RH was further decreased to 60%, the magnitude of the voltage fluctuations increased and then gradually decreased on continued operation at 60% RH. The low ion conduction at low moisture is responsible for this decreasing trend. The RH was then gradually increased again to 80% and 100%. During the final period of operation at 100% RH, the AEMFC voltage and HFR had returned to its initial level (with a slight increase in voltage noise). However, these results do show that the ZIL‐MEA is resilient to RH cycling, which is essential to the stable and efficient transport of ions between the polymer electrolyte components in the cell (Figure 5C,E and Figure S15, Supporting Information). Therefore, compare with the reported conventional strategy (increasing physical friction of the interface) for AEMFCs,^[^ ^59^ ^]^ our thermally triggered interlocking strategy can better prevent the interfacial delamination in the MEA at high temperature and RH cycling during fuel cell test.

## 3 Conclusions

We present a thermally triggered covalent‐locking strategy to fabricate a durable MEA in AEMFCs. SEM images and 3D reconstruction using computed micro‐X‐ray tomography confirm thermal inter‐crosslinking between the AEM and CLs, which creates a stable 3D‐zipped interface. Ex situ evaluation of interfacial bonding strength shows that the 3D‐ZIL significantly improves the mechanical properties of the MEA. In situ fuel cell durability evaluations (constant current with and without gas humidity cycling) demonstrates that the 3D‐ZIL prevents interfacial delamination during AEMFC operation, thus maintaining sufficient ion and reactant mass transport in the MEA, even with high current density cell discharges. A competitive AEMFC peak power density of 1.50 W cm^−2^ is demonstrated at 70 °C and 0.2 MPa gas‐back pressures. Initial durability evaluations show that the ZIL‐MEA‐based AEMFC can maintain its performance for at least 120 h whilst discharging at 0.6 A cm^−2^ with the integrity of ZIL‐MEA. For ionomer research, some current state‐of‐the‐art high IEC ionomers, such as poly(aryl piperidinium)s, poly(aryl‐aryl piperidinium), side‐chain‐type polyfluorene, with excellent power density are actually faced with insufficient molecular weight or overlarge swelling. The present in situ crosslinked strategy can be a potential solution for these high IEC ionomers for durable AEMFCs or AEM water eletrolyzer (AEMWEs).

## 4 Experimental Section

### Preparation of BPPO

PPO (20 g, 167 mmol) was dissolved in chlorobenzene (400 mL), after which *N*‐bromosuccinimide (21 g, 117 mmol) and azodiisobutyronitrile (0.20 g) were added to the solution. The reaction solution was then heated to 130 °C for 4 h with stirring. Afterward, the mixture was poured into an excess of ethanol (2000 mL) to form a light‐brown fibrous precipitate of BPPO. The precipitates were washed with ethanol a further six times and then dried in a vacuum oven at 40 °C for 48 h.

### Preparation of TMAQPPO

BPPO (2.0 g) was dissolved in NMP (30 mL) at room temperature. The resulting solution of BPPO was slowly added (drop by drop) to a stirred solution of TMHDA (5 equiv.) in NMP (30 mL). After stirring at room temperature for 24 h, the TMAQPPO powder was precipitated into ether. The resulting TMAQPPO powder was recovered by filtration and washed with ether a further three times, before being dried under vacuum at room temperature for 24 h.^[^ ^44^ ^]^

### Preparation of VBBQPPO

VBC (6.0 mL) was added to a solution of TMAQPPO (2.0 g) in NMP (30 mL). After being stirred for 24 h at room temperature, the crude VBBQPPO powder was precipitated into ether and recovered by filtration. The slightly yellow powder was then washed with ether for at least a further three times and dried under a vacuum at room temperature for 48 h.^[^ ^45^ ^]^

### Preparation of DMAQA

Bromoethane (4.2 mL) in ethanol (30 mL) was added dropwise to a stirred solution of TMHDA (12 mL) in ethanol (150 mL). After stirring for 24 h at room temperature, ethanol was evaporated in vacuum followed by the washing of the residue by ether for several times. Then, the white precipitate was added to acetone (50 mL) after filtration. As acetone was removed by filtration, the product was collected as white powder. Recrystallization from acetone/ether mixture was used to obtain pure DMAQA.

### Preparation of BQPPO

BPPO (2.0 g) was dissolved in NMP (30 mL) at room temperature. DMAQA (2.0 g) in NMP (20 mL) was slowly added (drop by drop) to a stirred solution of BPPO solution. After stirring for 24 h at room temperature, the mixture was poured into the excess ether, and BQPPO powder was collected by filtration. Finally, the BQPPO powder was washed with ether several times.^[^ ^46^ ^]^

### Formation of CBBQPPO

CBBQPPO was formed via the thermally initiated intercrosslinking of VBBQPPO (after deposition in the catalyst layers of the electrodes) by heating the electrodes to 60 °C for 2 h.

### Fabrication of VBBQPPO AEM, BQPPO AEM, and CBBQPPO AEM

VBBQPPO powder (1.0 g) was dissolved in NMP (15 mL), after which the solution was cast onto a clean glass plate and treated at 30 °C for 48 h to fabricate the noncrosslinked VBBQPPO AEMs. Similarly, BQPPO or VBBQPPO solution (1.0 g BQPPO or VBBQPPO in 15 mL NMP) was cast onto a glass plate followed by heating at 60 °C for 24 h to fabricate the BQPPO AEM or crosslinked CBBQPPO AEM. The resulting tough and transparent AEMs were peeled off with a controlled thickness of 15 ± 1 µm.

### Fabrication of MEAs

The MEAs were prepared by the GDE method. 10.2 mg Pt/C catalyst (HISPEC 9100, cathode) or PtRu/C catalyst (HISPEC 10 000, anode) (both 60% wt metal content, produced by Johnson Matthey Co.) were ultrasonically mixed with 100 mg deionized water, 400 mg 2‐propanol, and 51.1 mg AEI ethanol solution (5 wt%, VBBQPPO or BQPPO) to obtain well‐dispersed ink (catalyst:AEI = 4:1). The AEMs and AEIs (VBBQPPO or BQPPO) in the CLs have same IEC value (2.00 ± 0.02 mmol g^–1^), water uptake, and swelling ratio. The resulting catalyst inks were inkjet‐printed onto the GDEs (Toray TGP‐H‐060) to controlled loadings of 0.5 mg_metal_ cm^–2^ of metal loading (12.25 cm^2^ of electrode area for both anode and cathode) to obtain the relevant GDEs. The prepared GDEs and AEM were then immersed in aqueous 1 m NaOH solution to convert them to the OH^−^ form. The resulting GDEs were then placed on each side (PtRu/C at anode and Pt/C at the cathode) of the AEM to fabricate the MEA.

### Fabrication of ZIL‐MEA

ZIL‐MEA was fabricated by assembling VBBPPO‐AEM with VBBPPO‐GDEs first and then in situ crosslinking during break‐in procedure under 60 °C for 2 h. In contrast, M‐MEA was fabricated by assembling precrosslinked CBBPPO‐AEM with CBBPPO‐GDEs. M‐MEA‐B was fabricated by assembling uncrosslinkable BQPPO‐AEM with BQPPO ‐GDEs as another control.

### Electron‐Microscopy Analyses

A Hitachi‐7700 (working at 100 kV) was used to record TEM micrographs of the catalyst powders. The surface morphology of the catalyst layer (anode or cathode) and the cross‐section sandwich structure (catalyst layer and membrane) were characterized by a cold field environmental scanning electron microscopy (Hitachi, SU8220).

### Energy‐Dispersive X‐ray Spectrometry

To analyze the element distribution of nitrogen, carbon, platinum, and ruthenium in two MEAs, energy dispersive X‐ray spectrometry was collected using an X‐max 80 (Oxford Aztec).

### X‐ray Photoelectron Spectroscopy

Powder X‐ray diffraction patterns of catalyst samples were conducted on X‐ray photoelectron spectroscopy (ESCALAB 250 System) using Al K*α* excitation radiation (1486.6 eV).

### Atomic Force Microscopy

The surface morphology of VBBQPPO@Pt/C catalyst was further characterized using a Bruker high‐resolution AFM powered by PeakForce Tapping (Bruker Dimension icon with ScanAsyst).

### 3D Characterization of the MEAs

The computed micro‐X‐ray tomography (micro‐CT) tests were performed on a lab‐based X‐ray CT system (Zeiss Xradia Versa 520, Carl Zeiss XRM). Samples were mounted on the holder with an aluminum tube as the adapter. This was rotated horizontally by ±180°, pausing at discrete angles to collect 2D projection images, which were then combined to produce a 3D reconstruction of the sample's volume dataset. The ZIL‐MEA and M‐MEA samples (post durability test) were imaged with scanning resolutions of 0.7 µm per voxel, respectively, using a scanning energy of 50 kV per 4 W.

### Measurement of the Interfacial Bonding Strength

The interfacial bonding strength for both ZIL‐MEA and M‐MEA based laminates were characterized in air using a Q800 dynamic mechanical analyzer (DMA, TA Instruments) with a stretch rate of 0.5 N min^–1^. The assembly involved two GDEs (20 × 5 mm^2^) where a quarter of each was laminated at 60 °C and 5 MPa for 2 h. The test was conducted by pulling the two unlaminated sides in opposite directions. For each sample, the tests were repeated five times at 0% RH state, and the curves presented above average curves. The interfacial bonding strength of the interface is defined as the force required to separate the interface normalized by the specimen width (5 mm).

### Single‐Cell Performance Test

Single‐cell AEMFCs were tested using an 850e fuel cell test station (Scribner Associates, USA) in a galvanostatic mode with cell temperatures of 60 to 70 °C. To fully convert VBBQPPO in the MEA to the thermally crosslinked CBBQPPO form, the cell temperature was increased slowly from 30 to 60 °C over 1 h and held at 60 °C for 2 h with N_2_ gas feeds (100% RH, 0.5 L min^−1^) being supplied to both anode and cathode. After this MEA curing stage, the gases were switched to H_2_ and O_2_ gas (100% RH, 0.5 L min^−1^, anode and cathode, respectively) followed by an electrochemical activation stage (potentiostatic discharge at 0.50 V cell voltage and 60 °C until the current density stabilized for at least 30 min). After the break‐in procedure, the cell voltage at each controlled current density was recorded at different temperatures (60, 65, or 70 °C) or RH (60%, 80%, or 100% RH both in anode and cathode sides—these RHs were calculated from the gas dew points and cell temperature) with no back‐pressurization of the gases being used. High frequency impedances (HFR) at each current density were recorded at a frequency of 1000 Hz during AEMFC testing.

### Durability Test

The durability test was conducted at 70 °C with a flow rate of 0.5 L min^−1^ for both H_2_ and O_2_ at RH = 100% and with no backpressure. The operating voltages were recorded as a function of time at 70 °C with the cell held at 0.6 A cm^−2^ constant current density galvanostatic discharge.

### The RH Cycling Evaluation

The RH cycling evaluation was conducted at 70 °C with a flow rate of 0.5 L min^−1^ for both H_2_ and O_2_ with no backpressure with changes in the RH after every 10 h. The operating voltages were recorded as a function of time at 70 °C with the cell held at 0.6 A cm^−2^ constant current density galvanostatic discharge.

### Electrochemical AC Impedance Spectroscopy Analysis

EIS analysis is a well‐established experimental technique which can in situ elucidate polarization effects in the fuel cell using AC stimuli. Simulation of the EIS data can further quantify the deconvoluted capacitive and resistive contributions of both the membrane and interfaces in the single fuel cell (Table S3, Supporting Information), including prediction of the change in the morphology of the MEAs. Herein, the EIS analysis for the MEAs was performed for a single AEMFC containing the MEA under test, a pair of gaskets, and a pair of graphite blocks. Flow rates for both H_2_ and N_2_ gases (at the anode and cathode, respectively) were supplied at a constant 0.5 L min^–1^. Temperatures for the cell and gases were controlled at 70 °C. All the testing parameters such as gas flow rates and testing temperature were controlled using the Scribner 850e fuel cell test station. The EIS analysis was performed by an externally connected Autolab workstation (PGSTAT 302N, Metrohm, The Netherlands) controlled by Nova 2.1.2 software. Under potentiostatic EIS testing mode, a small sinusoidal potential perturbation (10 mV amplitude, *f* range 0.01–100 kHz) was applied between the cathode and anode plates, and the AC current response was measured. Impedance data was presented as Nyquist plots and were also simulated using ZSimpWin electrochemical analysis software (PAR Inc., USA). The electrical equivalent circuit (EEC) model selected was based on the possible physical replica comprising membrane and GDE. The best fitted results for each EEC parameter are presented in Table S3, Supporting Information.

## Conflict of Interest

The authors declare no conflict of interest.

## Authors Contribution

X.L. and X.L.G. contributed equally to this work. L.W., J.R.V., and T.W.X. conceived the project. L.W., T.W.X., and X.L. designed the experiments. X.L. and X.L.G. prepared the MEAs and conducted corresponding tests. X.L.G. and Y.B.H. helped with ionomers preparation. M.A.S. and M.X. helped with EIS testing. F.M.S. helped with sample preparation for SEM. R.B.‐S. helped with XPS and fuel cell testing. J.J.Z. and W.S.Y. prepared the membranes, collected and analyzed the data. Z.J.G. helped with AFM testing. C.P.W. and W.J.S. helped with membrane preparation and conducted TEM and SEM testing. X.L. and X.L.G. wrote the original draft. L.W., J.R.V., and T.W.X. further wrote and revised the manuscript. All authors contributed to the data analysis.

## Supporting information

Supporting Information

Videos S1

Videos S2

## Data Availability

Research data are not shared.
