# Supplementary material for: 3D‐Zipped Interface: In Situ Covalent‐Locking for High Performance of Anion Exchange Membrane Fuel Cells
Source: Adv Sci (Weinh). 2021 Oct 11;8(22):2102637. doi: 10.1002/advs.202102637 (PMC8596103; doi:10.1002/advs.202102637)
Supplement: Supplementary file 1 — Supporting Information [file ADVS-8-2102637-s002.pdf]

© 2021 Wiley-VCH GmbH

ADVANCED SCIENCE
Open Access

Supporting Information

for *Adv. Sci.*, DOI: 10.1002/advs.202102637

3D-Zipped Interface: In-situ Covalent-locking for High Performance of Anion Exchange Membrane Fuel Cell

Xian Liang, Xiaolin Ge, Yubin He, Mai Xu, Muhammad A. Shehzad, Fangmeng Sheng, Rachida Bance-Soualhi, Jianjun Zhang, Weisheng Yu, Zijuan Ge, Chengpeng Wei, Wanjie Song, Jinlan Peng, John R. Varcoe, Liang Wu,* and Tongwen Xu**

Supporting Information

3D-Zipped Interface: In Situ Covalent-Locking for High Performance of Anion Exchange Membrane Fuel Cells

Xian Liang†, Xiaolin Ge†, Yubin He, Mai Xu, Muhammad A. Shehzad, Fangmeng Sheng, Rachida Bance-Soualhi, Jianjun Zhang, Weisheng Yu, Zijuan Ge, Chengpeng Wei, Wanjie Song, Jinlan Peng, John R. Varcoe, Liang Wu,* and Tongwen Xu**

Materials

Poly(2,6-dimethyl-1,4-phenylene oxide) (PPO) was kindly supplied by Tianwei Membrane Company (Shandong, P. R. China). N-Bromosuccinimide (NBS), azobis-isobutyronitrile (AIBN), *N,N,N',N'*-tetramethyl-1,6-hexanediamine (TMHDA), 4-vinylbenzyl chloride (VBC), N-methylpyrrolidolone (NMP), ether, chlorobenzene, ethanol, methanol, sodium hydroxide (NaOH) were all used as purchased. The commercial catalysts were manufactured by Johnson Matthey. The commercial Pt/C catalyst is 60 % wt. of ~3 nm Pt nanoparticles on Vulcan XC-72 carbon support (HISPEC 9100). And the commercial Pt-Ru/C catalyst is 40 % by wt. of ~3 nm Pt nanoparticles and 20 % by wt. of ~3 nm Ru nanoparticles on Vulcan XC-72 carbon support (HISPEC 10000). Deionized water (DI water) was used throughout.

Ionomer structure characterization

Nuclear Magnetic Resonance (NMR) spectroscopy: ^{1}H NMR spectra were recorded using a Bruker 510 instrument (400 MHz for ^{1}H). Chemical shifts were reported in ppm relative to the signals corresponding to the residual non-deuterated protons in NMR solvents ($CDCl_3$: δ 7.26 ppm, CD_3OD: δ 3.31 ppm), whereas the internal reference was tetramethylsilane (TMS).

Fourier transform infrared (FT-IR) spectroscopy: FT-IR spectra were collected using a NICOLET iS10 of Thermo Fisher Scientific. The anionomer was measured when cast in the membrane form only. A sample of AEM was fixed between two CaF_2 optical plates followed

by natural drying in the atmosphere. The device was heated from 30 °C to 70 °C in four stages, with each 10 °C ramp taking 10 min and with each temperature being maintained for 60 min, during which the infrared absorbance spectra were recorded (2 min per measurement).

Characterization of AEMs

Water uptake and swelling ratio for AEMs: A sample of CBBQPPO or BQPPO AEM (10 × 40 mm) with a given mass was submerged in DI water for 24 hours at room temperature, followed by taken out and removed excess water on the membrane surface with tissue paper. Then, the weight and length of the hydrated membranes were measured quickly (denoted as W_w and L_d). Afterward, water uptake (*WU*) and swelling ratio (*SR*) was calculated as follows:

$$WU = \frac{W_w - W_d}{W_d} \times 100\%$$

$$SR = \frac{L_w - L_d}{L_d} \times 100\%$$

Where L_w and L_d are the length of the sample in hydrated and dehydrated conditions separately. W_w and W_d were defined as the mass of the sample in hydrated and dehydrated conditions, respectively.

Ion exchange capacity for ionomer and AEMs: Ion exchange capacity (IEC) of the prepared ionomer and AEMs was measured in the membrane form by the Mohr method. The membrane sample was soked in NaCl aqueous solution (1.0 M) at room temperature for 24 h, then washed several times with DI water and dried. The mass of the dehydrated sample (Cl^- form) was recorded and denoted as W_{cl^-}. Afterwards, Cl^- was released from the the membrane by immersion in Na_2SO_4 aqueous solution (1.0 M) for another 24 hours. Finally, the immersion solution was titrated with an aqueous $AgNO_3$ solution (0.1 M) employing K_2CrO_4 as indicator. The amount of $AgNO_3$ solution consumed during titration was recorded as V_{Ag+}. IEC was calculated as follows:

$$IEC(mmol/g) = \frac{V_{Ag^+}(mL) \times 0.1 mol/L}{W_{cl^-}(g)}$$

Hydroxide Conductivity: Hydroxide conductivity (OH^- conductivity) of the AEMs was measured in-plane using impedance spectroscopy with a standard four-point probe technique employing an Auto lab Zahner Zennium E (Germany) in galvanostatic mode and with an AC current amplitude of 0.01 mA and a frequency range of 1 MHz to 100 Hz. The AEM samples (OH^- form) were set into a Teflon cell and in contact with two current collecting electrodes and two potential sensing electrodes (the distance between the potential sensing electrodes was 1 cm). Then, the cell was quickly immersed in deionized water. Afterwards, the impedance spectrum was collected. The ionic conductivity (σ) was calculated according to the following equation:

$$\sigma = \frac{L}{RWd}$$

where L is the distance between potential-sensing electrodes (1 cm), R is the membrane resistance, d and W are the thickness and width of the AEM samples at the corresponding temperature respectively.

Alkaline stabilities estimation for AEMs: CBBQPPO or BQPPO AEM samples were soaked in a NaOH aqueous solution (1.0 M) at 70 ^{o}C for different time, followed by immersion in DI water and wash frequently for 48 h to remove the residual NaOH. Afterwards, OH^- conductivities of the samples were again measured as a function of time.

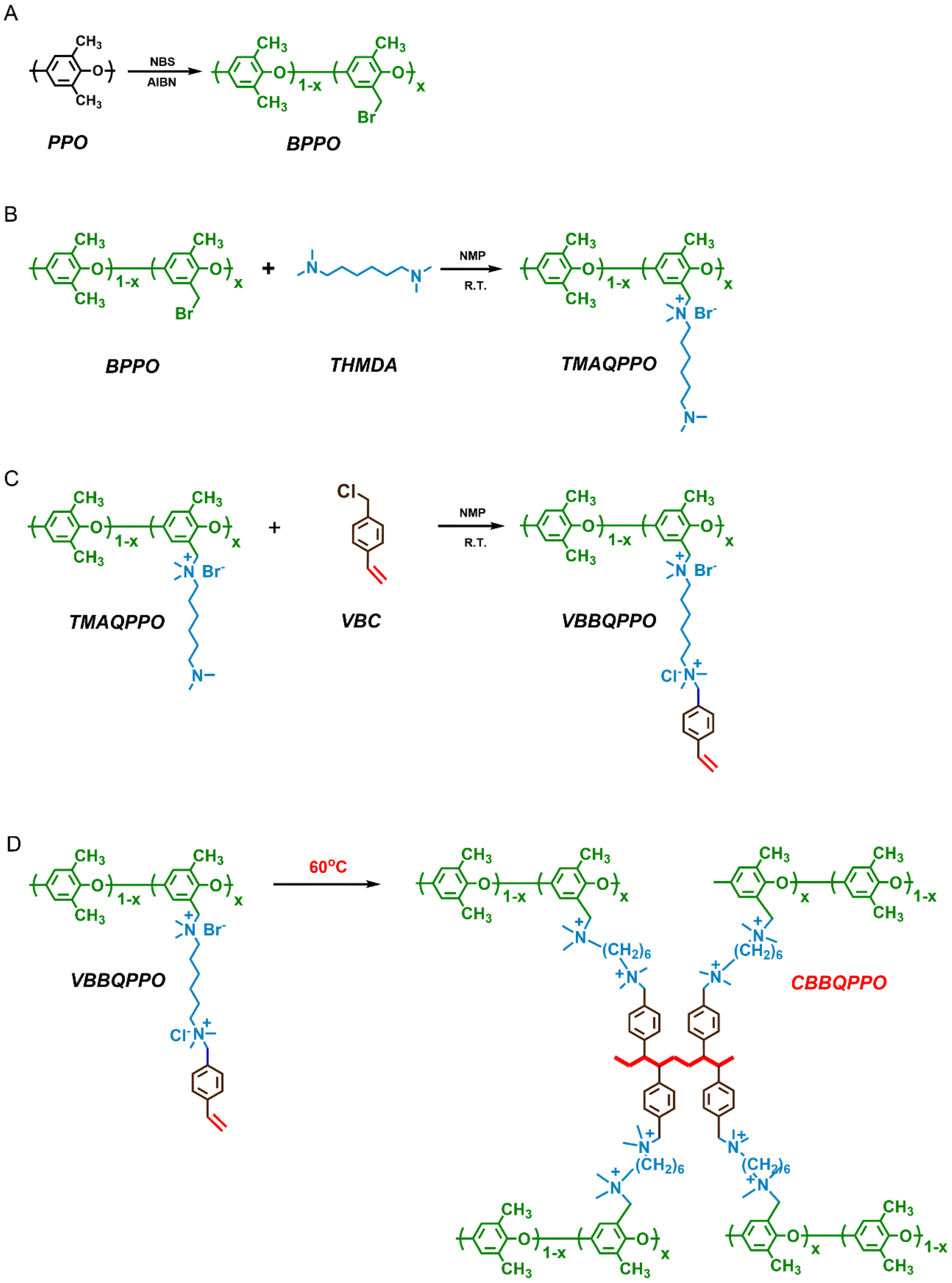

Scheme S1 Synthesis of BPPO, TMAQPPO, and VBBQPPO, along with the crosslinking process to form CBBQPPO. [55-56]

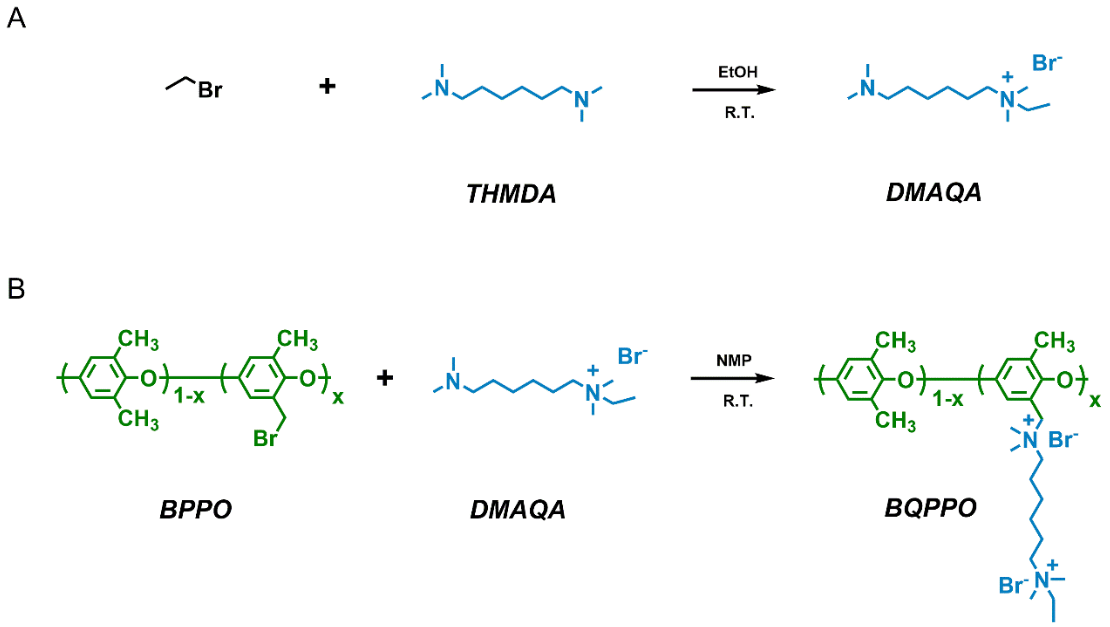

Scheme S2 Synthesis of BQPPO via the Menshutkin reaction between BPPO and DMAQA, comparing with CBBQPPO.[57]

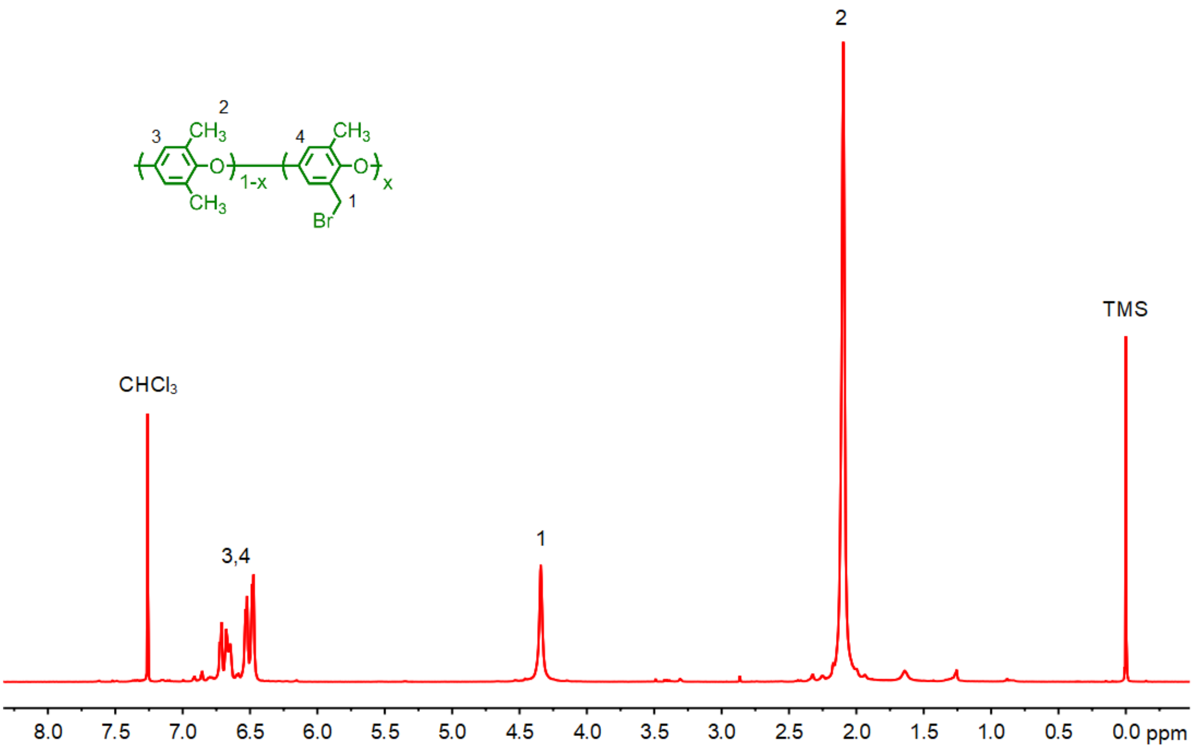

Figure S1 ^{1}H NMR spectrum of BPPO (400 MHz, $CDCl_3$, 298 K).

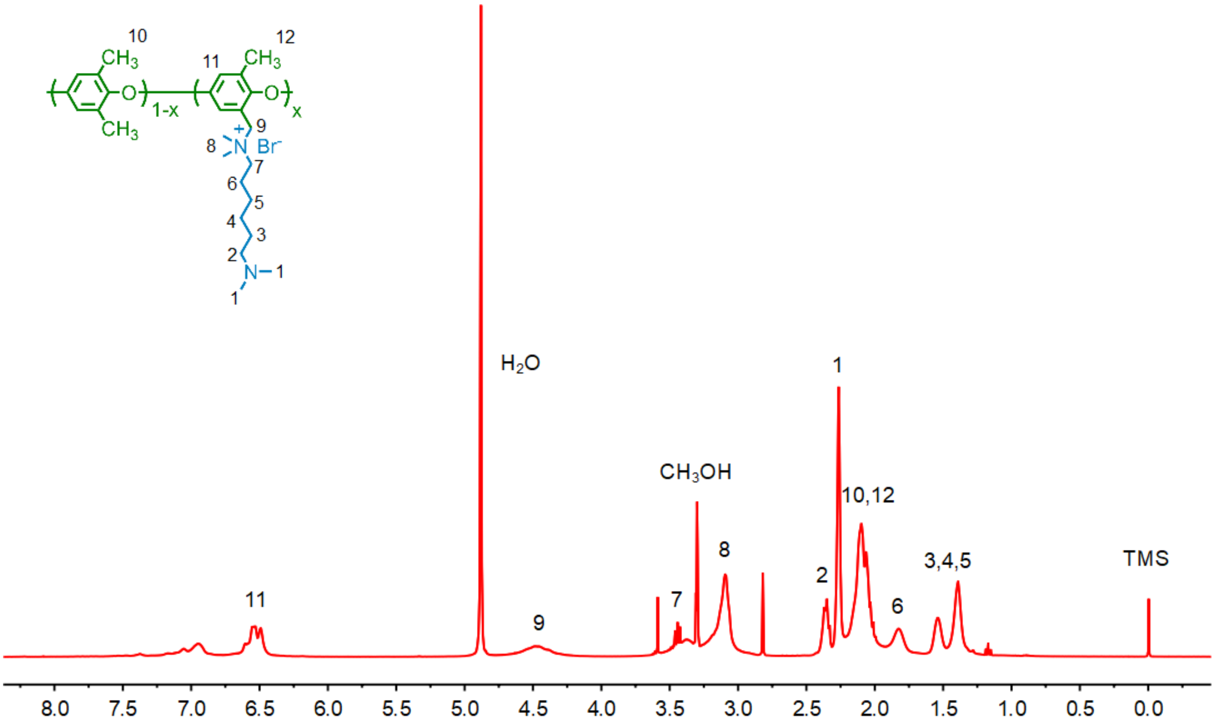

Figure S2 ^{1}H NMR spectrum of TMAQPPO (400 MHz, CD_3OD, 298 K).

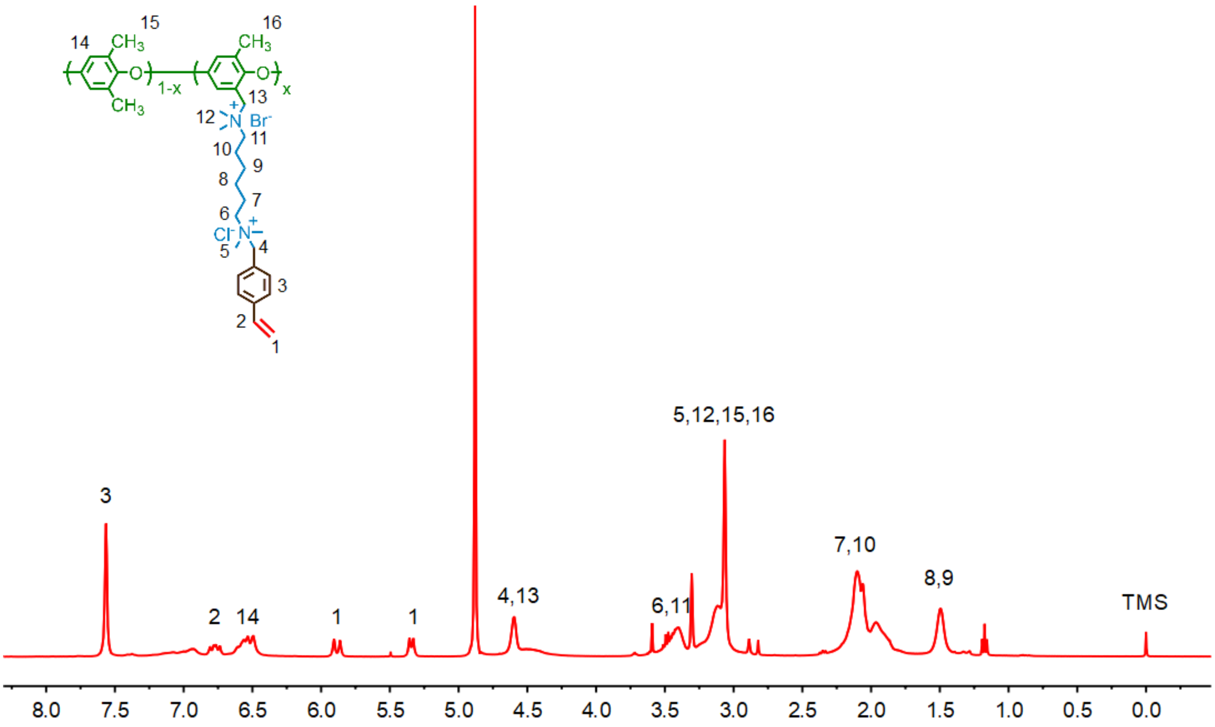

Figure S3 ^{1}H NMR spectrum of VBBQPPO (400 MHz, CD_3OD, 298 K).

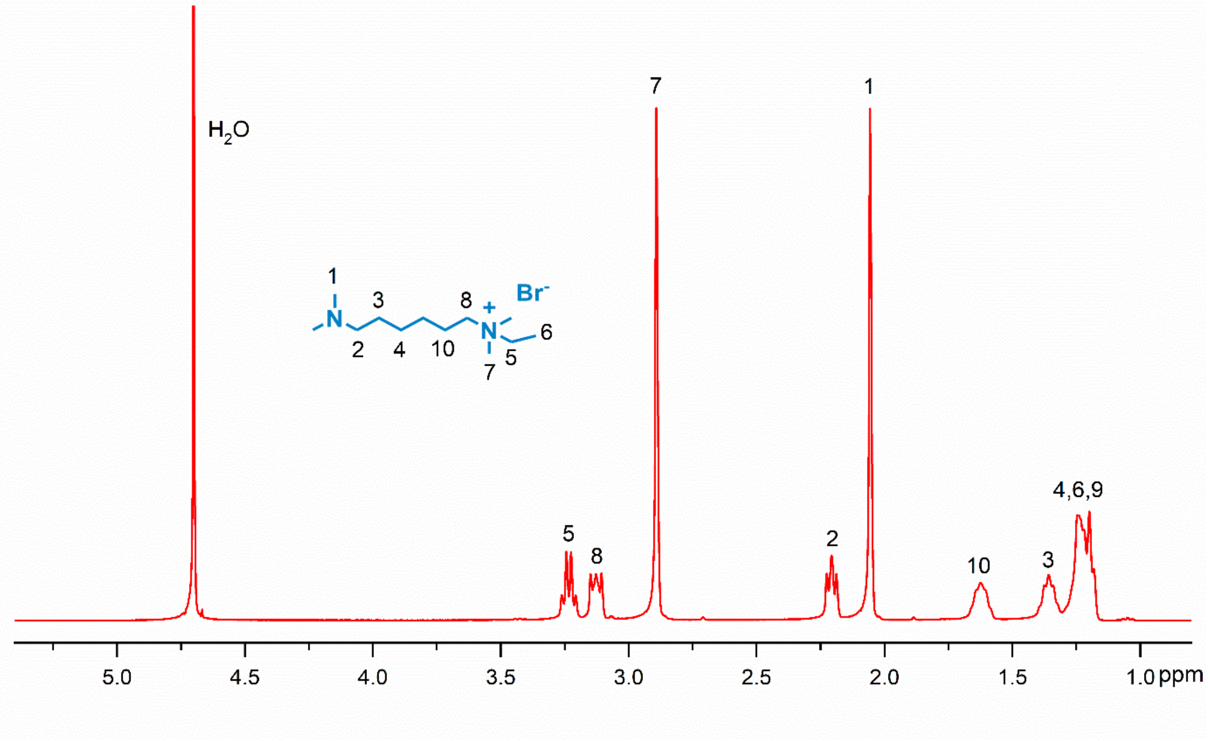

Figure S4 ^{1}H NMR spectrum of DMAQA (400 MHz, D_2O, 298 K).

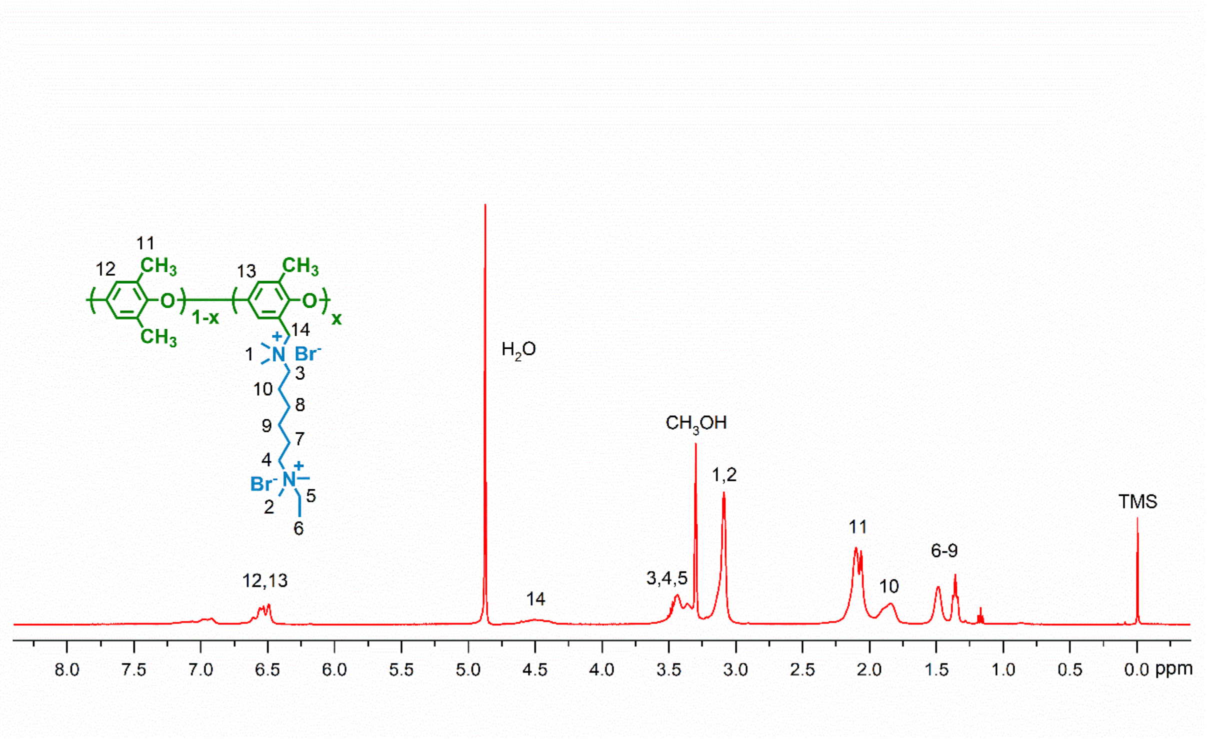

Figure S5 ^{1}H NMR spectrum of BQPPO (400 MHz, D_2O, 298 K).

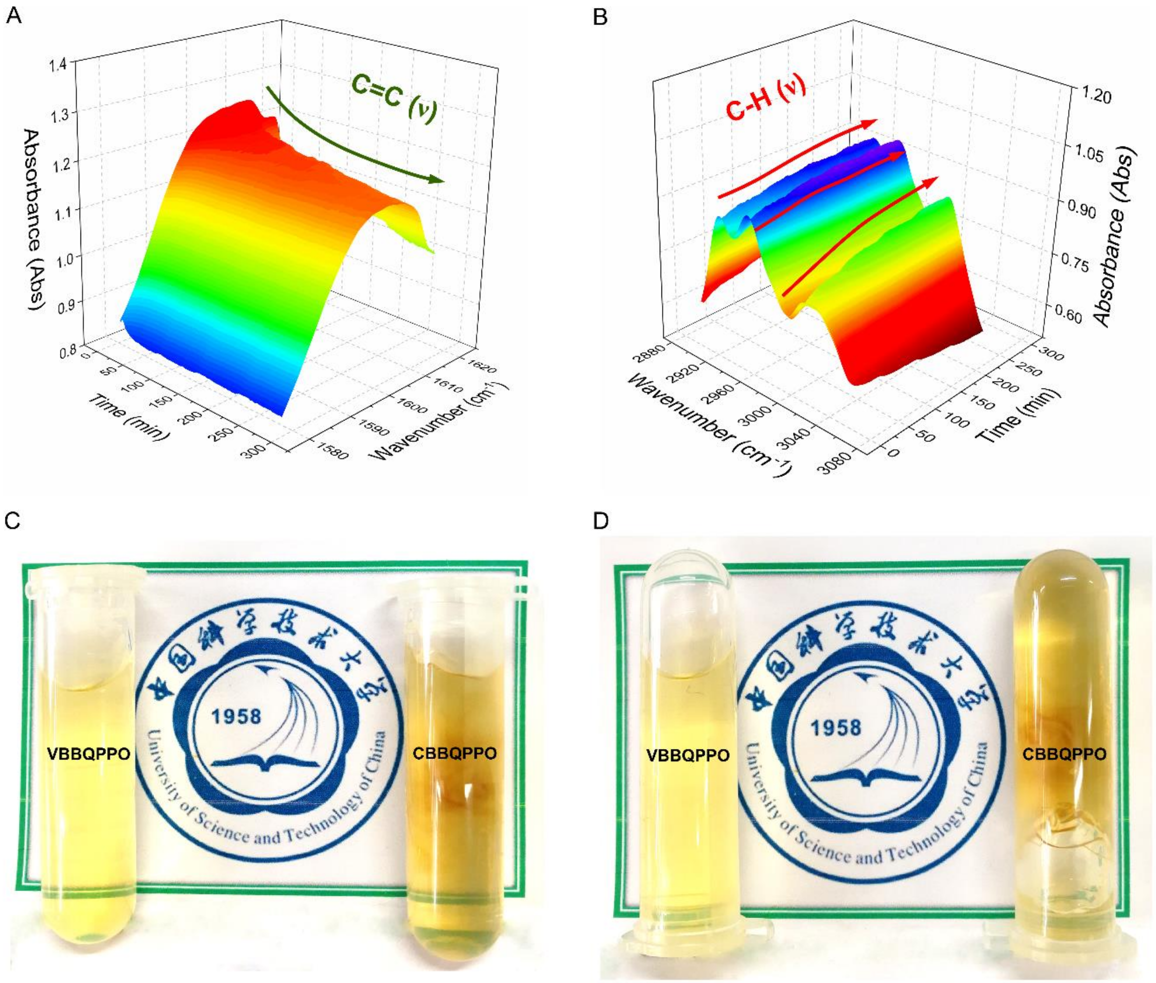

Figure S6 The thermally-initiated inter-crosslinking reaction converting VBBQPPO into cross-linked CBBQPPO. (A,B) The time-dependent *in-situ* FTIR spectroscopy of VBBQPPO (immobilized between two CaF_2 optical plates) recorded with heating to 70 ^oC at a heating rate of 1 °C min^{-1}; (C,D) The solubility of VBBQPPO and CBBQPPO membranes in NMP.

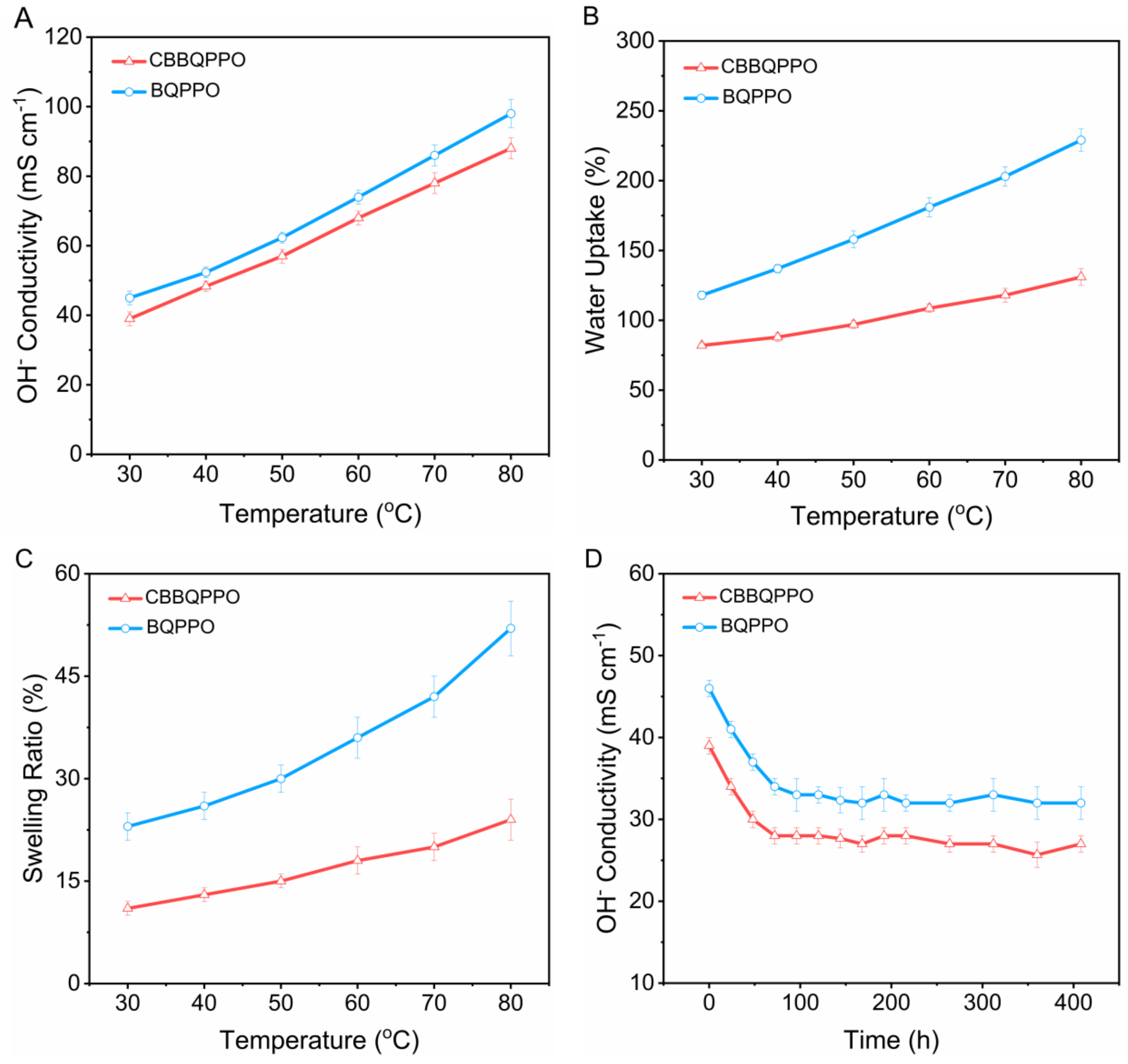

Figure S7 The OH^- conductivity (A), water uptake (B) and swelling ratio (C) of CBBQPPO and BQPPO AEMs as a function of temperature; (D) The hydroxide stability of crosslinked CBBQPPO and BQPPO membranes which immersed in 1.0 M NaOH at 70 ^{o}C.

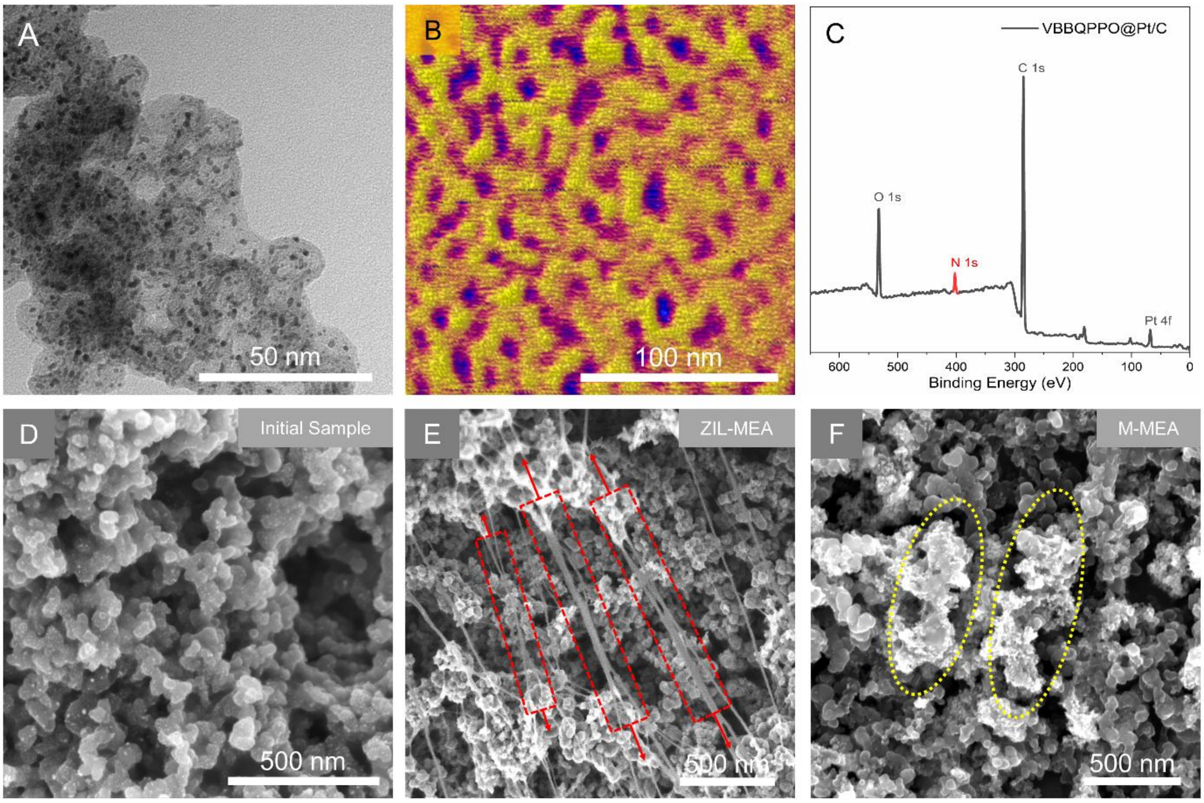

Figure S8 (A) TEM image of the VBBQPPO@Pt/C NPs; (B) AFM image of Pt/C nanoparticles on the surface of a VBBQPPO@Pt/C CL (deposited on a GDE); (C) Powder XPS spectrum of VBBQPPO@Pt/C catalyst. The surface SEM images of the CLs close to the membrane in the MEAs: (D) The initial surface of fresh-prepared VBBQPPO@Pt/C CL used in both the M-MEA and ZIL-MEA; (E) CBBQPPO Pt/C CL surface of the ZIL-MEA after the shear test, with highly deformed anionomers fibers; (F) CBBQPPO Pt/C CL surface of the M-MEA after the shear test.

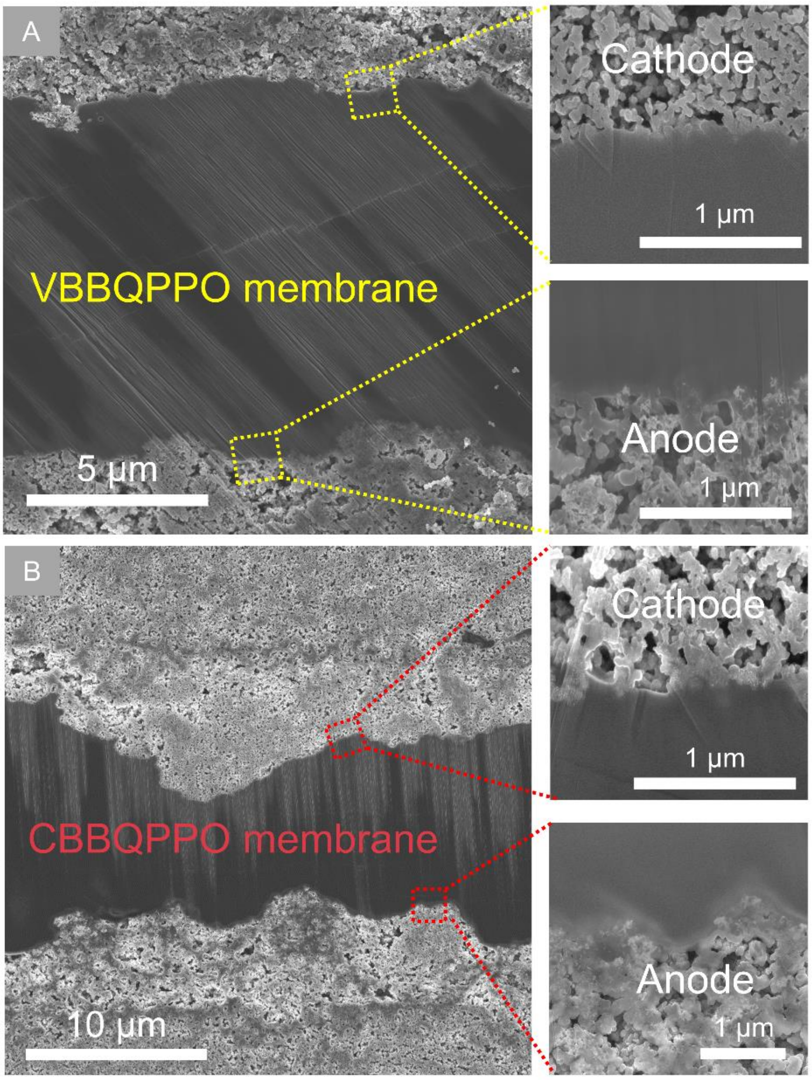

Figure S9 SEM cross-sectional images of the MEAs: (A) After lamination at room temperature, (B) After thermally-triggered covalent-crosslinking between the AEM and CLs.

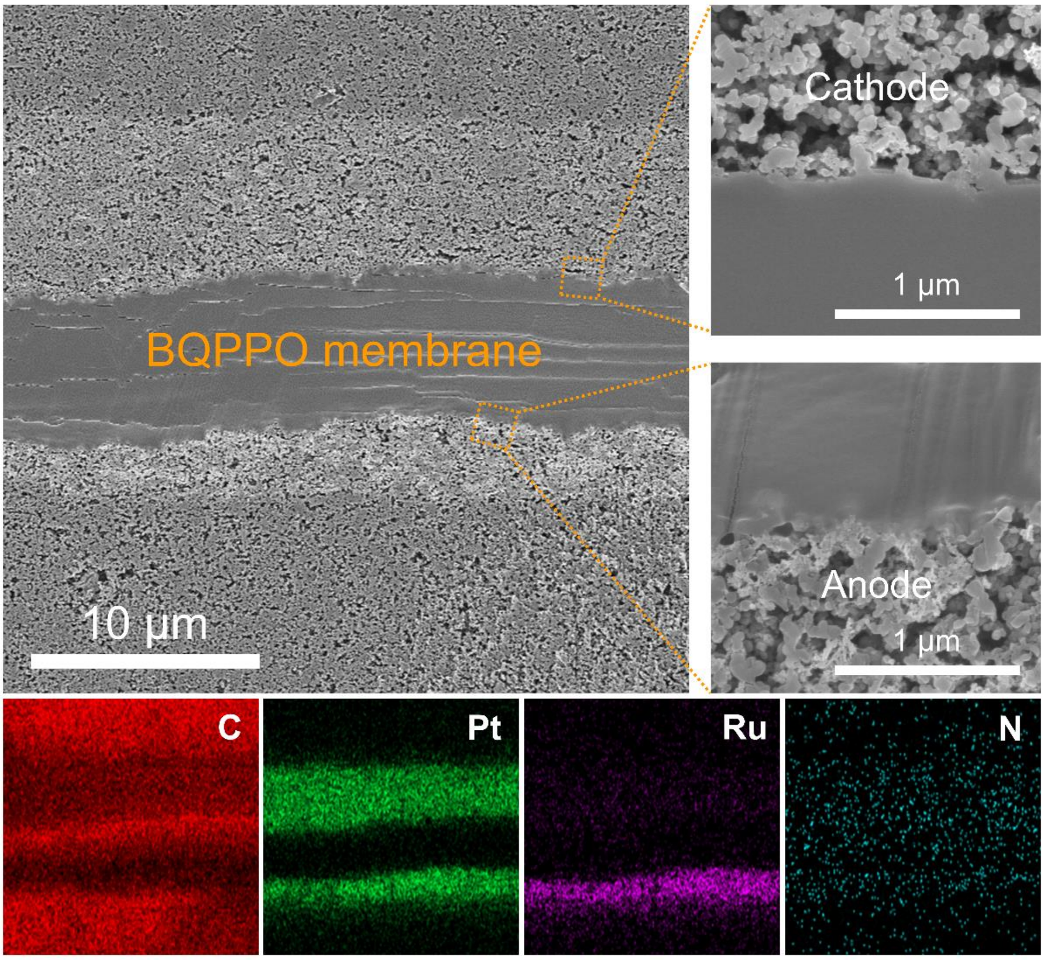

Figure S10 Cross-section SEM images coupled with EDX of the initial M-MEA-B.

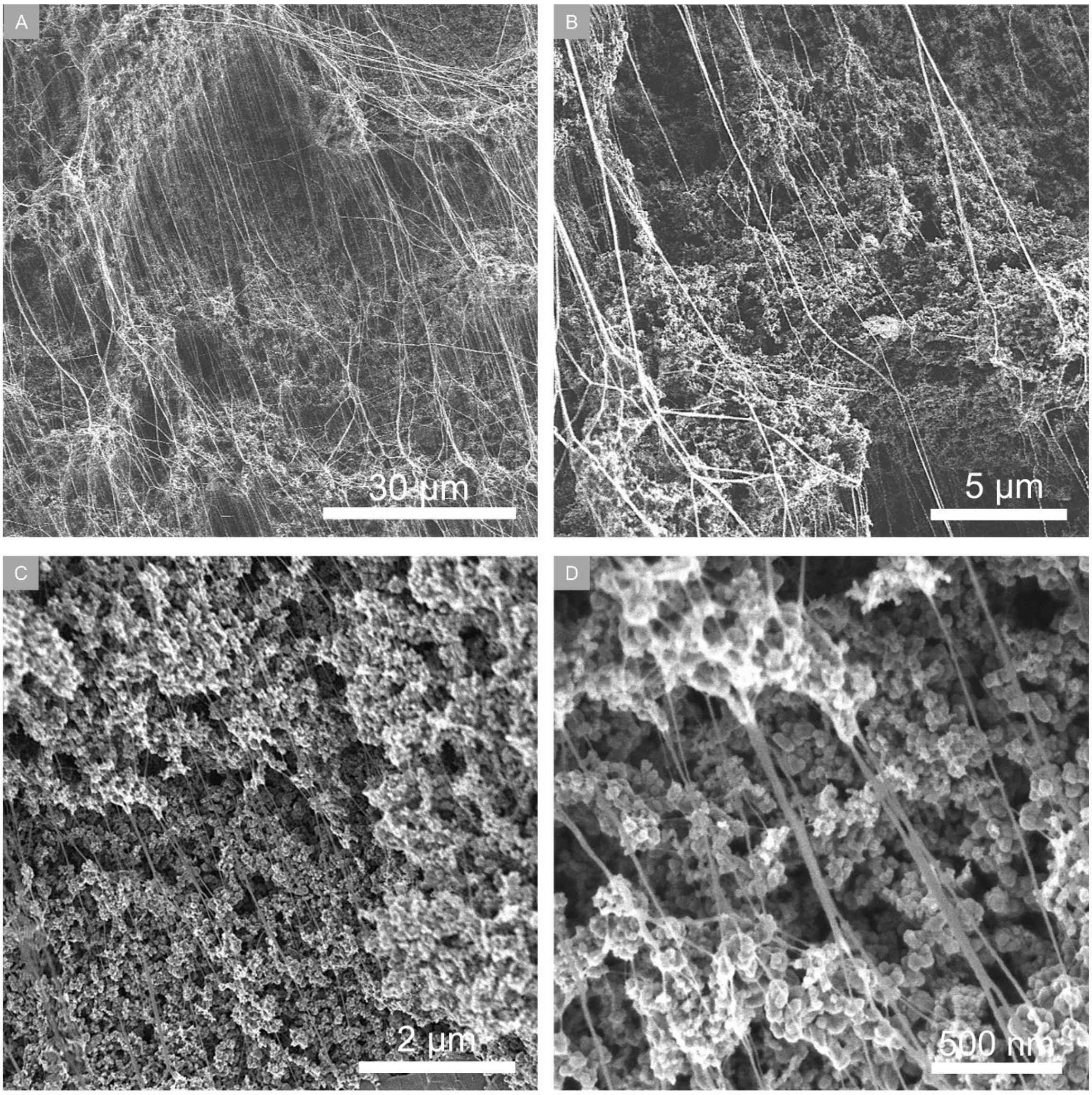

Figure S11 Cross-section SEM images of CBBQPPO Pt/C CL surface of the ZIL-MEA after the shear tests (recorded with increasing magnification).

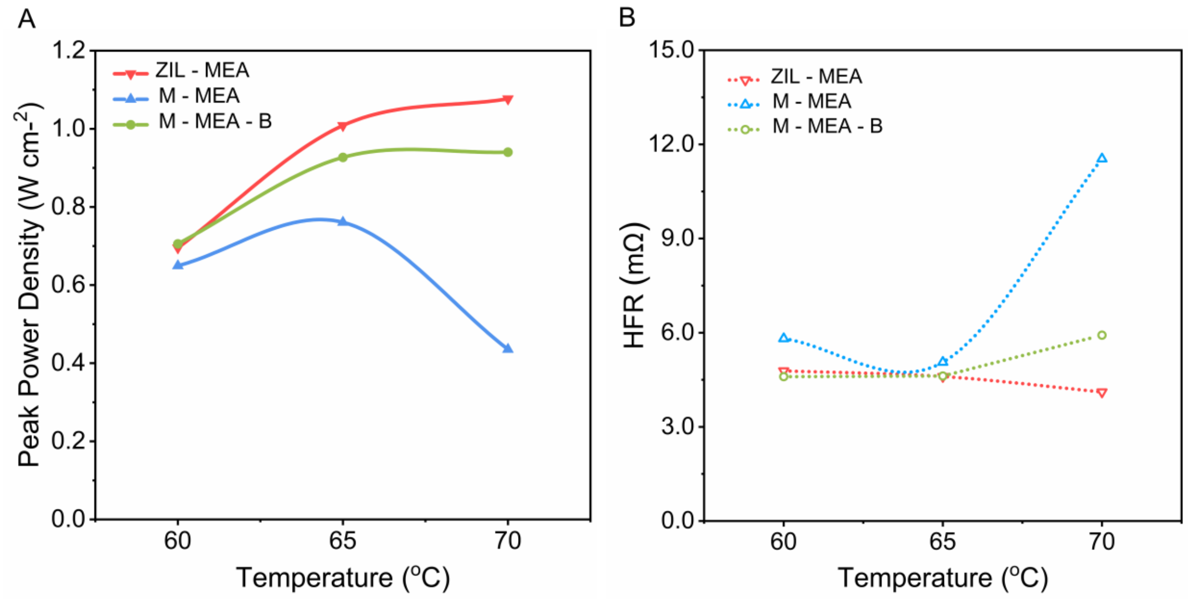

Figure S12 Peak power density and high frequency resistance (HFR) comparison between ZIL-MEA, M-MEA and M-MEA-B during the AEMFC tests (stack area = 12.25 cm^2) at 60, 65 and 70 °C. The connecting lines are a guide to the eye only.

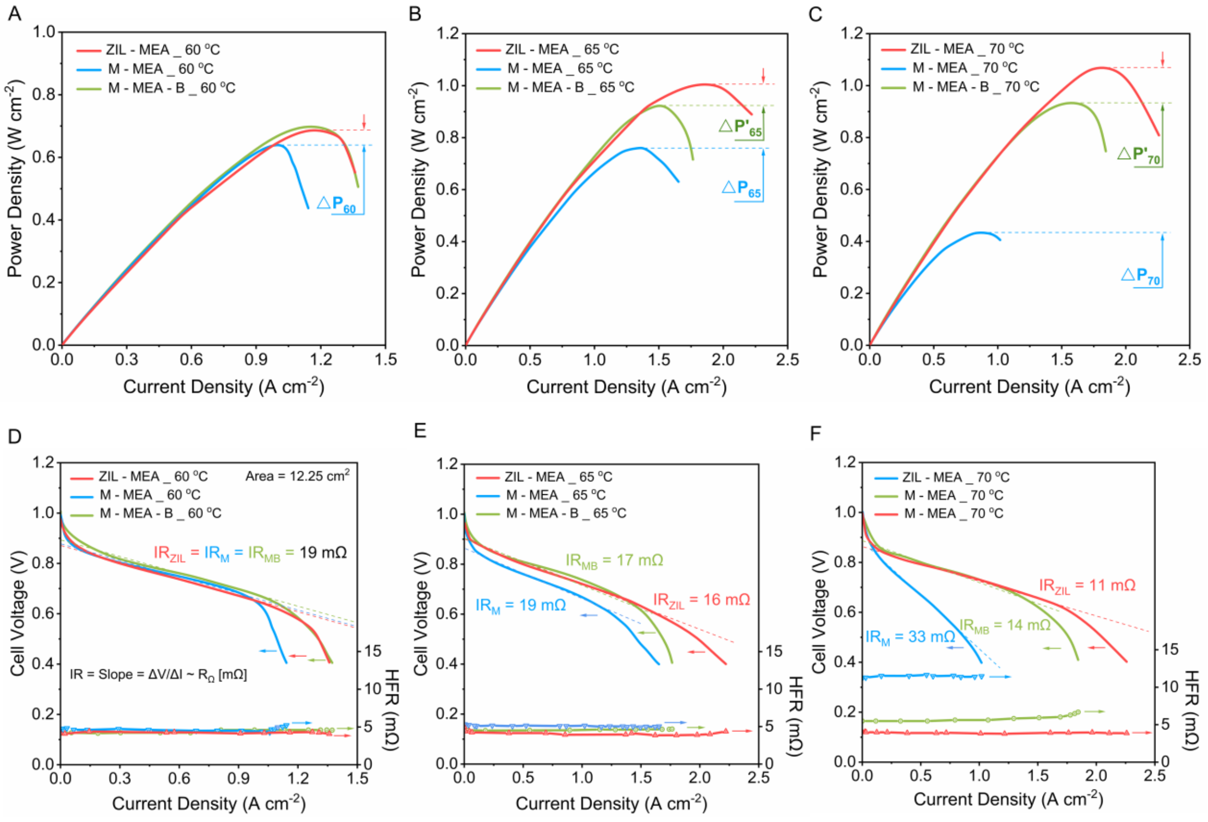

Figure S13 H_2/O_2 single-cell AEMFC performance data (PPD, HFRs, and IR values for the Ohmic region of the curves) for ZIL-MEA, M-MEA and M-MEA -B at 60, 65, 70 °C.

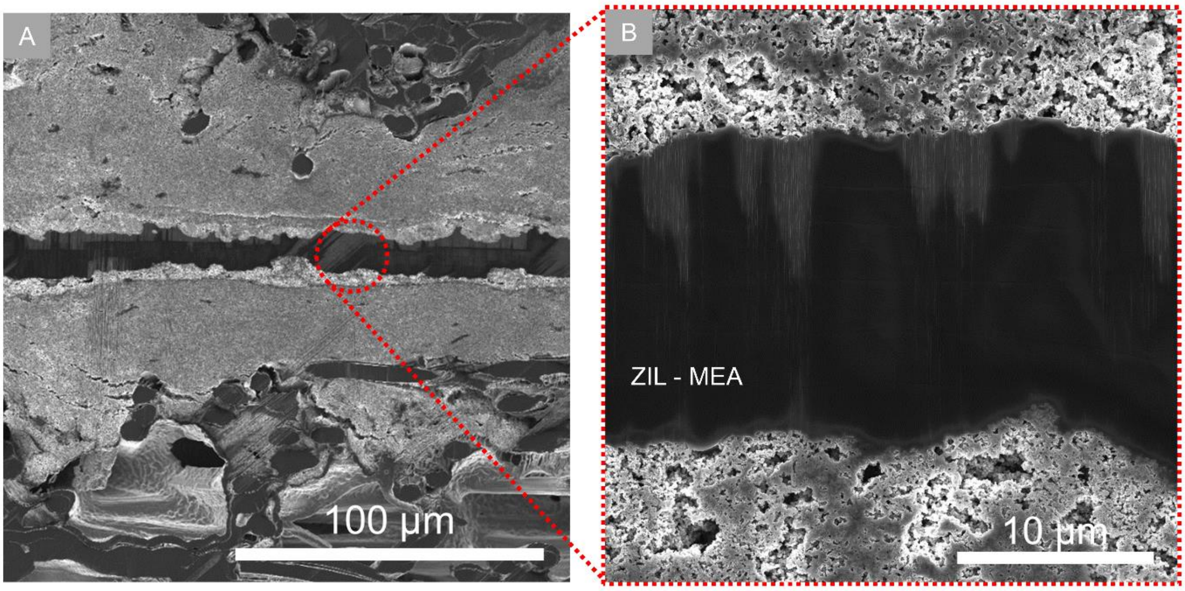

Figure S14 Cross-sectional SEM images of the ZIL-MEA after AEMFC durability tesing at 70 °C.

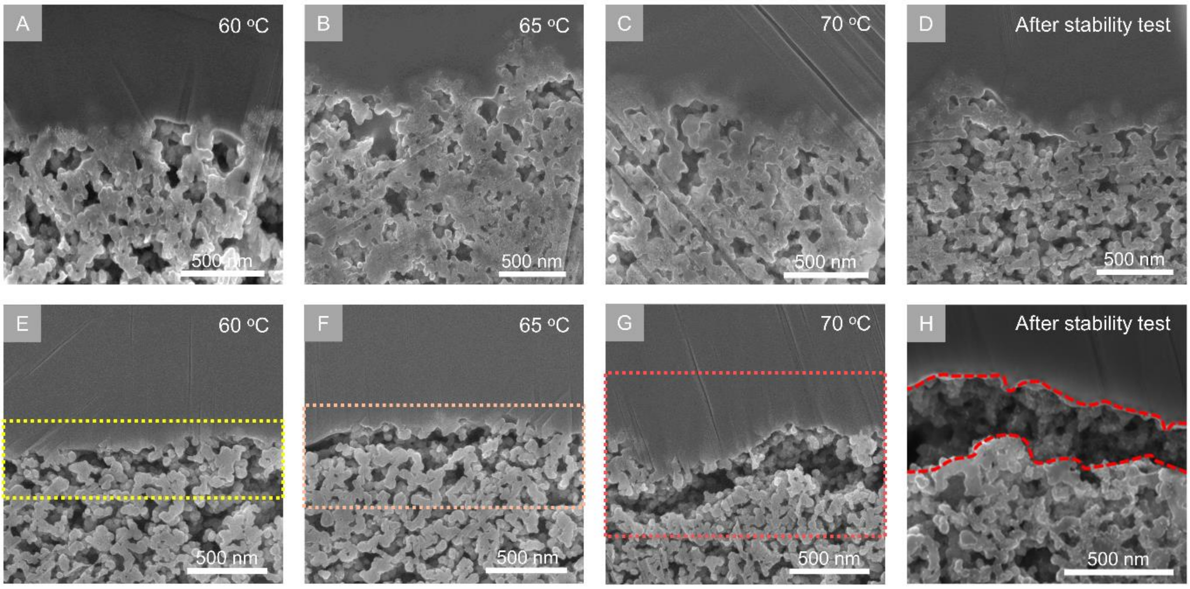

Figure S15 Cross-sectional SEM images of cathode side of both the ZIL-MEA and M-MEA after AEMFC testing and durability testing at the indicated temperatures. Dashed lines represent changes in the interface between the AEM and CL.

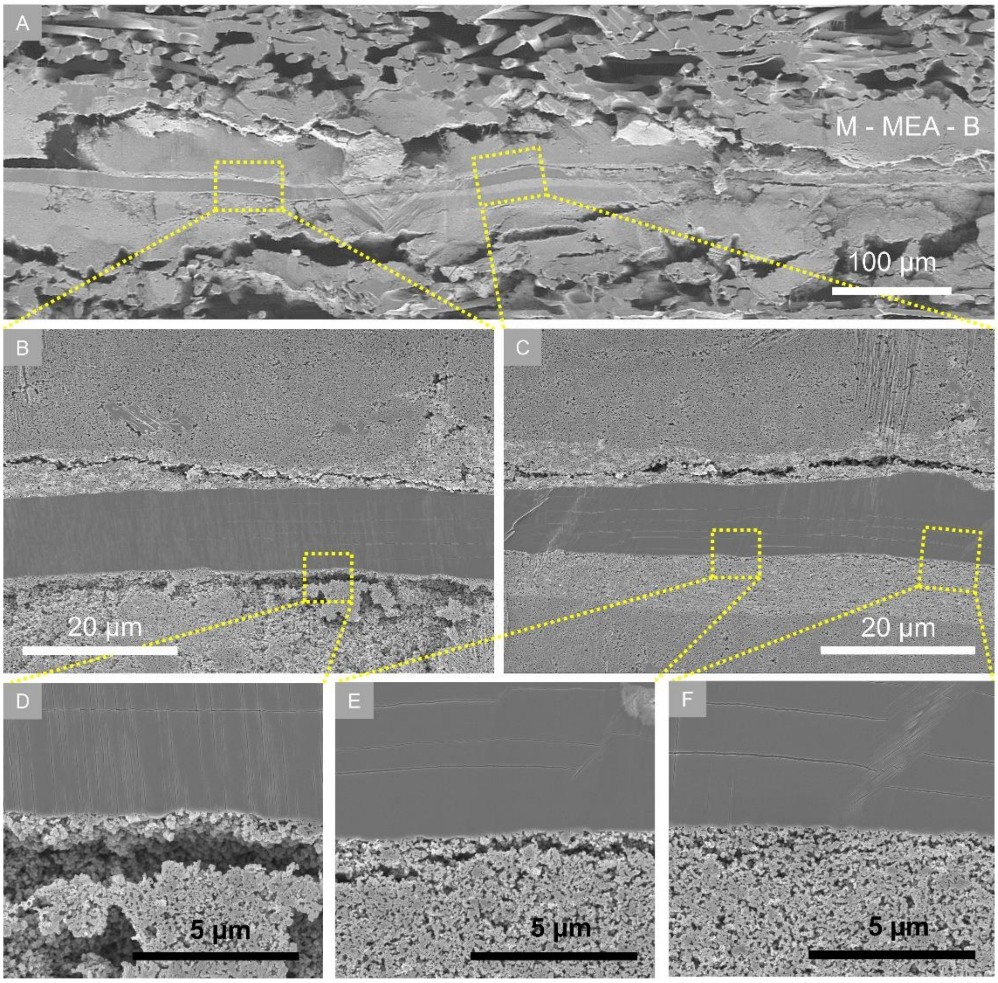

Figure S16 Cross-sectional SEM images of the M-MEA-B after AEMFC durability testing at 70 °C.

Table S1 Fuel cell data (PPD and durability) of recently reported AEIs or AEMs for H_2/O_2 AEMFCs

AEIs or AEMs	Peak Power Density (W cm^{-2})	Life Time (h)	Voltage Decay Rate (μV h^{-1})	Constant Current Density (A cm^{-2})	Cell Temp (°C)	A/C Temp (°C)	Flow Rate in Durability Test (L min^{-1})		Catalyst		A/C Metal Loading mg cm^{-2}	Literatures
							Anode	Cathode	Anode	Cathode		
ZIL-MEA	1.50	120	~ 667	0.6	70	70/70	0.5	0.5	PtRu/C	Pt/C	0.5/0.5	Our Work
M-MEA-B	0.94	45	~ 11333	0.6	70	70/70	0.5	0.5	PtRu/C	Pt/C	0.5/0.5	Our Work
M-MEA	0.76	8	~ 57500	0.6	70	70/70	0.5	0.5	PtRu/C	Pt/C	0.5/0.5	Our Work
PPO-BTMA	0.08	8.5	~ 35294	0.1	60	60/60	0.2	0.2	Pt/C	Pt/C	0.5/0.5	36
PPO-SCPi	0.11	8.5	~ 29411	0.1	60	60/60	0.2	0.2	Pt/C	Pt/C	0.5/0.5	18
SDQEO	0.29	12	~ 20583	0.2	60	60/60	0.1	0.1	Pt/C	Pt/C	0.5/0.5	19
PPO-ASU	0.12	40	-	-	60	60/60	0.5	0.5	Pt/C	Pt/C	0.5/0.5	20
PPO-c-PVP	0.17	48	~ 2083	0.1	80	80/80	0.1	0.1	Pt/C	Pt/C	0.5/0.5	21
PPO-SEBS	0.40	300	~ 1933	0.2	60	58/58	0.2	0.4	Pt/C	Pt/C	0.5/0.5	22
CPFBP-TQA	0.12	24	~ 1792	0.1	80	80/80	0.1	0.1	Pt/C	Pt/C	-	23
PES–NS	0.11	24	~ 833	0.1	60	60/60	0.1	0.1	Pt/C	Pt/C	-	24
TQ-PDBA	0.16	48	~ 1667	0.1	80	80/80	0.12	0.12	Pt/C	Pt/C	0.6/0.6	25
QPC-TMA	1.61	50	~ 2727	0.6	60	58/59	0.8	1.0	PtRu/C	Pt/C	0.4/0.4	26
PDTP-25	2.58	100	~ 2000	0.4	80	80/80	0.2	0.2	PtRu/C	Pt/C	0.4/0.4	15
PBN-100Pip	0.40	120	~ 4083	0.2	80	80/80	0.2	0.2	Pt/C	Pt/C	-	27
TEA-o-BTN	1.50	120	~ 1583	0.6	80	80/80	2	0.3	Pt/C	Pt/C	0.6/0.6	28
QPAE/GO-(APTS-c-PTMA)	0.14	120	~ 1500	0.1	70	70/70	0.2	0.3	Pt/C	Pt/C	0.3/0.3	29
PX75-T50	0.73	400	~ 170	0.4	80	80/80	1.0	1.0	PtRu/C	Pt/C	0.5/0.5	30
HDPE-AEM	2.55	440	~ 136	0.6	70	68/70	1.0	1.0	Pt/C	Pt/C	0.6/0.6	13
StIm-based AEMs	0.71	670	~ 610	0.05	60	60/60	0.5	0.5	PtRu/C	Pt/C	0.71/0.53	31
FLN-55/FLN-	1.20	900	~ 422	0.6	80	80/80	1.4	0.3	PtRu/C	Pt/C	0.75/0.6	32
ETFE-g-poly (VBTMAC)	2.31	1000	32	0.6	65	65/65	1.0	1.0	PtRu/C	Pt/C	0.7/0.6	33
GT	3.2	2000	15	0.6	75	72/74	0.3	0.3	PtRu/C	Pt/C	0.7/0.6	16

"~" indicates that there is no exact value of voltage decay rate in the literatures, which is just approximately calculated from durability data with galvanostatic mode (voltage - time curves).

Table S2 List of physical parameters for the dry state AEMs for the interfacial bonding strength of analysis.

	ZIL-MEA	M-MEA-B	M-MEA
**Displacement / μm**	124.23	81.46	22.91
**Static Force / N**	6.50	4.96	3.97
**Bonding Strength / N mm^{-1}**	1.30	1.02	0.79
**Failure strain / %**	0.59	0.35	0.14

Table S3 Simulation electrical equivalent circuit (EEC) fits for the impedance spectra recorded with M-MEA and ZIL-MEA before and after AEMFC durability testing. The measured (msd) EIS Nyquist data in Figure 4b was simulated (sim) with a best-fit equivalent electrical circuit model (standard error < 0.5 %, $\chi^2 < 10^{-4}$), $LR_M(Q_{MGI}R_{MGI})(QcatRcat(Q_PR_P))$, where contribution of the electrically conductive fuel cell components (including electrodes) was modelled by the inductance element (L) and R_M represents the ohmic resistance of the AEM. The first circuit, $(Q_{MGI}R_{MGI})$ attributes the charge-storage (Q = constant phase element) and charge-transfer resistance (R) between the membrane-GDE interface (MGI). The second circuit, $(QcatRcat(Q_PR_P))$ represents the charge-storage and charge-transfer resistance values at the catalyst-polymer interface within the GDE. The Q_n is the index value ($0 < Q_n < 1$) of the constant phase element (Q).

EEC Parameters LR(QR)(QR(QR))		Za ZIL-MEA -after	Zi ZIL-MEA -initial	Ma M-MEA -after	Mi M-MEA -initial	Ma M-MEA-B -after	Mi M-MEA-B -initial
Error (%)		0.2361	0.2101	0.3731	0.2916	0.2062	0.3614
Chi Sq. value		8.163e-6	7.966e-6	3.858e-6	5.109e-6	4.987e-04	2.605e-04
L (H)		1.112e-7	1.144e-7	1.634e-7	1.784e-7	1.606e-5	1.259e-5
**R_M (mΩ)**		**43.4**	**40.9**	**79.4**	**48.3**	**71.8**	**41.9**
Q_{MGI}	Y_{MGI} (S cm^{-2} s^n)	0.6809	0.7339	0.8693	0.7837	0.671	0.766
	$Q_{n\text{-}MGI}$	0.8360	0.8425	0.8615	0.7599	0.8091	0.8255
**R_{MGI} (mΩ)**		**41.7**	**35.1**	**375.8**	**36.4**	**243.9**	**35.9**
Q_{Cat}	Y_{Cat} (S cm^{-2} s^n)	0.04543	0.05084	0.03709	0.03258	0.0341	0.049
	$Q_{n\text{-}Cat}$	0.6687	0.6772	0.6881	0.6815	0.658	0.668
**R_{Cat} (mΩ)**		**55.81**	**43.77**	**62.98**	**46.89**	**64.1**	**40.39**
Q_P	Y_P (S cm^{-2} s^n)	0.01958	0.01595	0.01794	0.02113	0.0184	0.0158
	$Q_{n\text{-}P}$	0.9005	0.9239	0.8441	0.7188	0.76	0.87
**R_P (mΩ)**		**19.2**	**14.4**	**272.9**	**64.9**	**217.4**	**50.2**

Y = Non-ideal capacitance
If Q = 1 then Y = Capacitance
